# Multi-omic cross-sectional cohort study of pre-malignant Barrett’s esophagus reveals early structural variation and retrotransposon activity

**DOI:** 10.1038/s41467-022-28237-4

**Published:** 2022-03-17

**Authors:** A. C. Katz-Summercorn, S. Jammula, A. Frangou, I. Peneva, M. O’Donovan, M. Tripathi, S. Malhotra, M. di Pietro, S. Abbas, G. Devonshire, W. Januszewicz, A. Blasko, K. Nowicki-Osuch, S. MacRae, A. Northrop, A. M. Redmond, D. C. Wedge, R. C. Fitzgerald

**Affiliations:** 1 Medical Research Council Cancer Unit, Hutchison/Medical Research Council Research Centre, University of Cambridge, Cambridge, CB2 0XZ UK; 2 Cancer Research UK Cambridge Institute, University of Cambridge, Robinson Way, Cambridge, CB2 0RE UK; 3 Wellcome Centre for Human Genetics, University of Oxford, Roosevelt Drive, Headington, Oxford, OX3 7BN UK; 4 NIHR Oxford Biomedical Research Centre, Oxford University Hospitals NHS Foundation Trust, John Radcliffe Hospital, Headley Way, Headington, Oxford, OX3 9DU UK; 5 Department of Histopathology, Addenbrooke’s Hospital, Cambridge, CB2 0QQ UK; 6 Manchester Cancer Research Centre, University of Manchester, Wilmslow Road, Manchester, M20 4GJ UK; 7 Present Address: Department of Gastroenterology, Hepatology and Clinical Oncology, Centre of Postgraduate Medical Education, Warsaw, Poland

**Keywords:** Cancer genomics, Cancer genomics, Oesophageal cancer, Genomic instability, Barrett oesophagus

## Abstract

Barrett’s esophagus is a pre-malignant lesion that can progress to esophageal adenocarcinoma. We perform a multi-omic analysis of pre-cancer samples from 146 patients with a range of outcomes, comprising 642 person years of follow-up. Whole genome sequencing reveals complex structural variants and LINE-1 retrotransposons, as well as known copy number changes, occurring even prior to dysplasia. The structural variant burden captures the most variance across the cohort and genomic profiles do not always match consensus clinical pathology dysplasia grades. Increasing structural variant burden is associated with: high levels of chromothripsis and breakage-fusion-bridge events; increased expression of genes related to cell cycle checkpoint, DNA repair and chromosomal instability; and epigenetic silencing of Wnt signalling and cell cycle genes. Timing analysis reveals molecular events triggering genomic instability with more clonal expansion in dysplastic samples. Overall genomic complexity occurs early in the Barrett’s natural history and may inform the potential for cancer beyond the clinically discernible phenotype.

## Abstract (web summary)

The alterations associated with progression from Barrett’s esophagus (BE) to esophageal adenocarcinoma are not fully characterised. Here, the authors perform a multi-omics analysis of a longitudinally-sampled BE patient cohort, identifying the impact of structural variants, including mobile elements, and the timing of molecular events during progression.

## Introduction

Epithelial cancer is typically preceded by a pre-malignant phase with pathologically-defined features. Understanding the steps underlying the transition from pre-invasive to invasive disease is the key to the development of early detection strategies^1,2^. Esophageal adenocarcinoma (EAC) is a poor prognosis cancer often preceded by a metaplastic precursor called Barrett’s esophagus (BE); the unresolved clinical challenge is to identify those patients with BE who are most at risk. To put this into perspective, BE is relatively common in the general population with a prevalence of 1–2%^3,4^ (up to 10% in those with reflux symptoms^5–7^), but fewer than 1% of these patients progress each year to cancer^8,9^. Despite this low progression rate, early diagnosis is highly clinically relevant for this disease because pre-invasive, dysplastic BE lesions can be cured with endoscopic resection and/or ablation. This outpatient-based treatment for the early disease is in stark contrast to the lengthy and invasive treatment regimens required for EAC, comprising of systemic chemotherapy, with or without radiotherapy and esophagectomy, with associated morbidity and poor 5-year survival^10^.

BE can progress to cancer gradually via histologically-defined, pre-invasive stages called low-grade dysplasia (LGD), high-grade dysplasia (HGD) and intramucosal carcinoma (IMC). This grading determines management but dysplasia can be missed due to sampling bias from random biopsy sampling at endoscopy, and it is a morphological diagnosis that is subjective. Furthermore, dysplasia does not adequately portray the underlying molecular features such that some patients appear to progress rapidly and unpredictably^11,12^. Molecular profiling has improved our understanding of the mutational processes and the driver gene landscape underlying EAC^13–16^. However, how early these processes are laid down is not yet fully understood, especially with regards to the large-scale chromosomal rearrangements and retrotransposon activity that dominate the EAC genome in comparison to the relative paucity of recurrent mutations in oncogenes^17–19^.

The majority of studies to date have focussed either on comparing BE sampled from adjacent to cancer^20–25^, or comparing indolent, non-dysplastic BE with cancer^26,27^. Where dysplasia has been considered, it has mostly been with very small numbers of patients^28,29^. When BE is sampled from adjacent to cancer, usually from the esophagectomy resection specimen, the epithelium is found to be highly mutated and heterogeneous even when compared to many other invasive cancers, irrespective of grade^21,22^. However, these samples represent the final stage in cancer evolution, and it is not known to what extent the adjacent BE samples recapitulate the earlier time points in the natural history of the disease.

Identifying cancer drivers has been a substantial challenge in this cancer type. Loss of *TP53* appears to be an early event^30–35^ although it is not yet certain whether *TP53* mutations are confined to the dysplastic grades or can be seen in non-dysplastic segments prior to progression to dysplasia^33,36^. Aside from *TP53* mutation, other driver gene mutations occur in low frequency^13,36^. It has been known for some time that copy number alterations (CNAs) increase in frequency during malignant progression and that these may be key for determining the natural history. For example, high-density SNP array studies and gene panels have shown CNAs to increase with progression, with the early loss of 9p21 (*CDKN2A*) and later loss of 17p (*TP53*)^32–34^. We recently used shallow whole-genome sequencing (0.4× WGS) from formalin-fixed paraffin-embedded (FFPE) tissues to obtain genome-wide copy number data and then developed a logistic regression model to identify regions of the genome with CNAs that could predict a patient as being high risk prior to progression to dysplasia^37^. However, whilst achieving our goal for identifying a clinically applicable biomarker, very shallow WGS allows the analysis of CNAs but does not have the resolution to show single nucleotide variants (SNVs) or allow detailed analysis of large-scale structural variants (SVs). Furthermore, in that study we relied on archival FFPE tissues ahead of progression to dysplasia or cancer and the availability and quality of material meant that we could not generate matched multi-omic data.

In this work, we aim to provide an unbiased, high resolution genome-wide and multi-omic assessment of the molecular landscape across the pathological grades of BE in order to better understand the determinants of dysplasia and cancer. Rather than interrogating the earliest time point in a patient’s history, here we focus on the highest grade of dysplasia that a patient manifests during extensive follow-up so that we could characterise the events marking different phenotypic disease stages between patients. We thus performed high depth (50×) WGS in fresh frozen tissues, which permits the analysis of SVs and mobile elements spanning non-coding regions, as well as whole transcriptome and methylation array data in a large cross-sectional cohort of 146 patients. We correlated the findings with detailed clinical and pathological data regarding the grade of disease and clinical trajectory. We demonstrate that progression is defined molecularly by increasing genomic instability, with a landscape defined by complex structural variants and increasing LINE-1 retrotransposon activity from indolent, non-dysplastic to progressed, dysplastic stages. The burden of structural variants is a gradual continuum, rather than a stepwise progression through the dysplasia grades, which generally differentiates indolent cases from those that have progressed to dysplasia. These data suggest that molecular features accumulate over time until the resulting genomic instability tips the balance to progression.

## Results

### Characteristics of the cohort

**Fig. 1:**
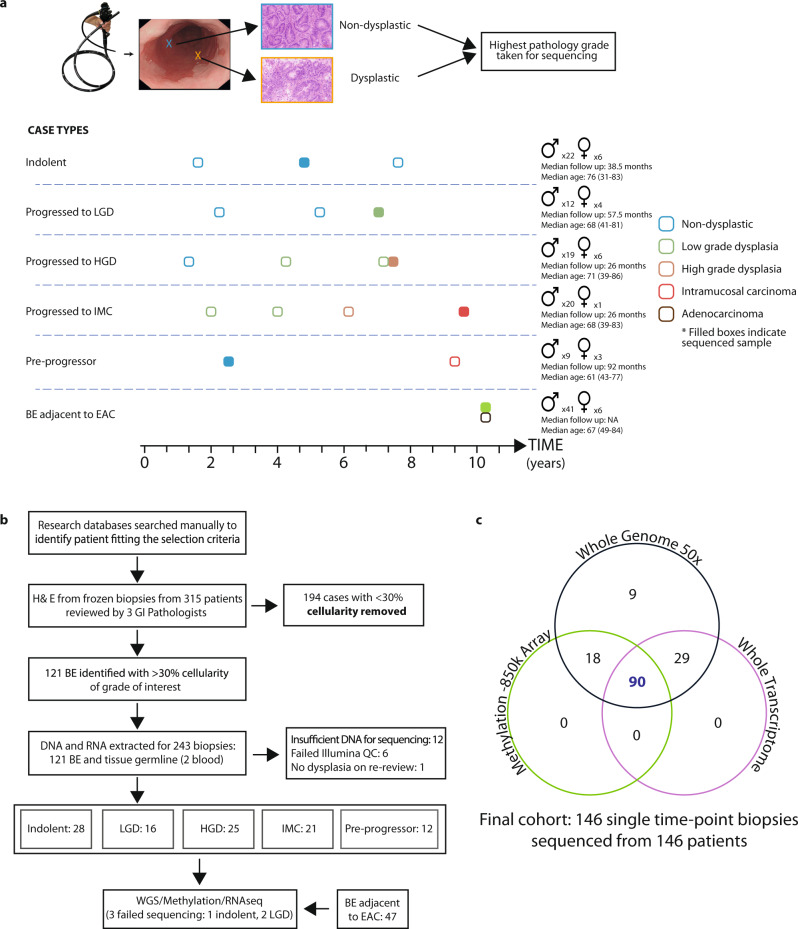
Study design and sequencing strategy. **a** Examples of representative cases selected for sequencing, with surveillance endoscopies over time and the highest grade at the time point detailed: indolent non-dysplastic; cases which progressed to low-grade dysplasia (LGD) high-grade dysplasia (HGD) or intramucosal carcinoma (IMC); cases sampled prior to progression to dysplasia; Barrett’s esophagus (BE) sampled from adjacent to esophageal adenocarcinoma, at the cancer time point. The highest available pathology grade frozen biopsy was used for sequencing. Examples of frozen biopsy histology are shown. Gender, median follow-ups after the sequenced sample and median age and range detailed. **b** Flow diagram detailing cohort creation. 149 high-cellularity, triple pathology reviewed, single-time point BE biopsies sequenced from 149 individual patients with different disease trajectories were sequenced. **c** For each patient, one biopsy underwent whole-genome sequencing (WGS) at 50×, whole-transcriptome sequencing and methylation with the EPIC 850k array, where sufficient material was available. Three samples were later excluded due to genomic mismatch or poor coverage, resulting in an analysed cohort of 146 patients. The Venn diagram shows the numbers of biopsies sequenced with each modality.

From our prospective surveillance study of >3000 pre-cancer patients, we identified 315 suitable patients who reflected the different grades of BE and dysplasia and for whom we had high-quality frozen samples and extensive follow-up data. Dysplastic samples were taken from the latest follow-up endoscopy and prior to any ablative therapy, thus representing each patient’s highest grade of disease. H&E staining and stringent pathology review of a frozen section allowed us to identify the most representative sample per case with a homogeneous grade of dysplasia and adequate cellularity for multi-omic sequencing (described in “Methods” section; Fig. 1a, b). We thus included 28 long-term, non-progressing, non-dysplastic patients with the indolent disease and 62 dysplastic cases comprised of 16 low-grade dysplasia patients, 25 high-grade dysplasia patients, and 21 intramucosal carcinomas (tumour in situ/T1a lesions confined to the mucosa) patients. In addition, we considered 47 patients with prevalent, visible BE adjacent to invasive EAC for comparison with other studies that have used this design^21,22^. In a sub-study, we also compared the non-dysplastic, indolent cases with a small cohort of 12 “pre-progression” cases, sampled at an early, non-dysplastic time point prior to progressing to dysplasia to inform an analysis of the relative timing of events. All biopsies underwent whole genomic (50×), transcriptomic and epigenomic (850k array) profiling according to material availability. Three samples were later excluded due to genomic mismatch or poor coverage, resulting in an analysed cohort of 146 patients: 134 main cohort plus 12 pre-progression cases (Fig. 1c).

The overall median length of follow-up was 73 months (range 0–258 months) giving a total of 642 person-years: median non-dysplastic follow-up 139 months (range 44–258); median dysplastic follow-up 57 months (range 0–249) (Table 1). A comparison of clinical variables showed a significantly higher rate of tobacco smoking (complete data for 81.8% of cohort) for patients who had progressed to dysplasia (LGD, HGD, and IMC), compared to those with non-dysplastic BE on an indolent disease course (81.3% vs 45.5%; *p*-value = 0.004, Fisher’s exact test). No other significant differences in patient characteristics were observed between the pathological grades of disease (indolent non-dysplastic vs dysplastic) or outcome (indolent vs pre-progressor), including notably male:female ratio and segment length. However, cases with prevalent BE found adjacent to cancer at their EAC diagnosis had a higher M:F ratio (Table 1).

**Table 1:** Cohort demographics and clinical features.

	Indolent, non-dysplastic	Dysplastic	BE adjacent to EAC	Pre-progressor	*p*-value indolent vs dysplastic	*p*-value indolent vs pre-progressor
Number of patients	28	62	47	12		
Patient dysplasia grade	ND	(LGD: 16, HGD: 25, IMC: 21)	NA	ND		
Time between ND biopsy and future dysplasia in months, median (range)	NA	NA	NA	42.5 (15–100)		
Gender, M:F	3.7:1	4.2:1	6.8:1	3:1	0.78	1
Male	22	50	41	9		
Female	6	12	6	3		
Age in years, median (range)	76 (31–83)	69 (39–86)	67 (49–84)	61 (43–77)	0.43	0.034^a^
BMI, median (range)	28.8 (23.2–43.9)	28.9 (13.5–40.9)	28.1 (20.3–38.8)	27.8 (24.6–34.8)	0.86	0.67
NA	4	2	9	1		
Maximum length BE segment in cm, median (range)	5 (2–12)	6 (0.5–18)	Not recorded	4.5 (3–14)	0.56	0.84
Ethnicity						
White British	26	55	36	10		
White other	1	1	1	2		
Pakistani	0	1	0	0		
NA	1	5	10	0		
Smoker						
Y, *n* (%)	10 (45.5%)	39 (81.3%)	30 (71.4%)	5 (62.5%)	0.004	0.68
N	12	9	12	3		
NA	6	14	5	4		
PPI						
Y, *n* (%)	26 (92.8%)	59 (96.7%)	31 (77.5%)	12 (100%)	0.59	1
N	2	2	9	0		
NA	0	1	7	0		
NSAID						
Y	10 (38.4%)	20 (32.7%)	6 (37.5%)	1 (8.3%)	0.63	0.12
N	16	41	10	11		
NA	2	1	31	0		
Surveillance pre-study sample in months, median (range)	109 (5–233)	3 (0–193)	NA	48 (0–141)		
Follow-up post-study sample in months, median (range)	38.5 (0–116)	35 (0–127)	NA	92 (26–119)		
Total patient surveillance in months, median (range)	139 (44–258)	57 (0–249)	NA	154 (50–248)		

Statistical comparisons were made between indolent and dysplastic, and indolent and pre-progression groups. There were significantly more smokers (*p*-value = 0.004) in the dysplastic vs. indolent groups. *p*-values calculated using Fisher’s exact test for categorical variables and *t*-test (two-sided) for continuous variables.

*BMI* body mass index, *PPI* proton pump inhibitor, *NSAID* non-steroidal anti-inflammatory drugs, *ND* non-dysplastic, *LGD* low-grade dysplasia, *HGD* high-grade dysplasia, *IMC* intramucosal carcinoma, *NA* not applicable, *%* given exclude NA patients.

^a^The only significantly different variable between the indolent and pre-progressor cases was the age, with the pre-progressor cases being younger. However, this was likely due to the requirement to use a sample from early in the follow-up for pre-progressor cases.

Surveillance in months, prior to the sequenced sample, and follow-up in months after the study sample are given separately.

### A molecular continuum defined by an increase in structural variant events

On analysing the main cohort in an unbiased fashion, without consideration of pathological grade, we found that the total number of SV events captured the most variance between samples (Supplementary Fig. S1a, b) and consequently for further comparison patients were ordered according to the total number of SV events (SV burden). In this ordering, we did not consider translocations, as most were driven by retrotransposon activity and showed wide variability, particularly in prevalent BE adjacent to EAC samples. The ordering by SV number, including translocations, is shown in Supplementary Fig. S1c.

**Fig. 2:**
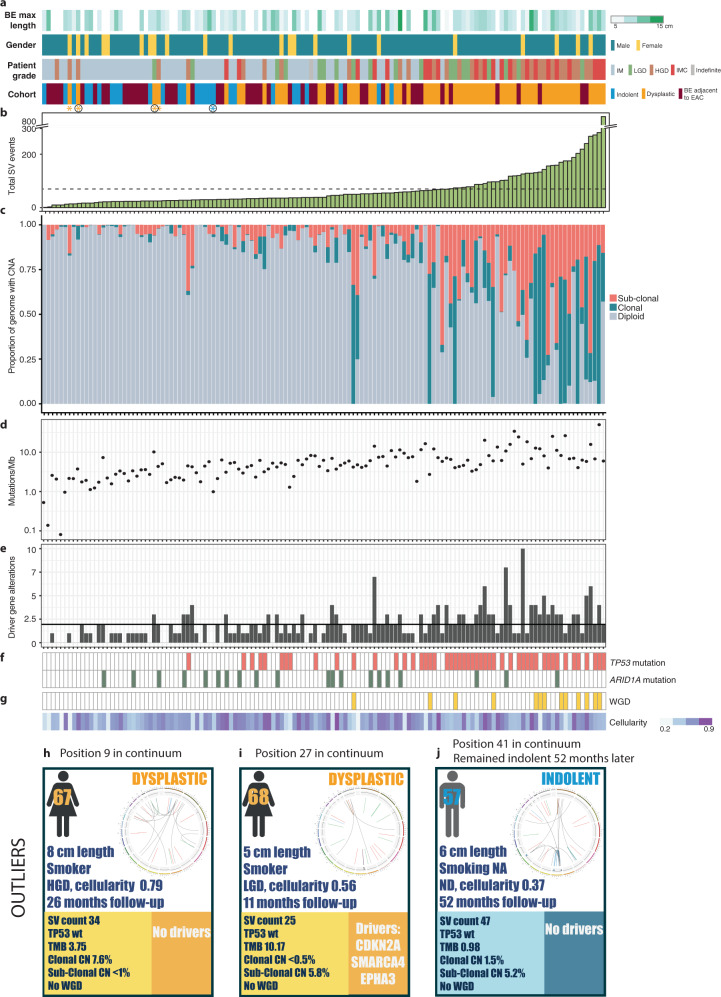
Genomic characteristics of the pre-cancer cohort. Samples are ordered by their total calculated numbers of structural variant (SV) events (*n* = 134). **a** Patient clinical features: maximum BE length (cm), gender, grade of patient at time of biopsy, patient group within the cohort. The patient grade indicates the highest grade which the patient had at the time point, but for the Barrett’s adjacent to cancer, for which the time point was cancer, the patient grade instead indicated the dysplasia status of the biopsy. Asterisks mark outlier cases discussed in more detail. **b** Total number of SV events per patient. Mean number of events (69.9) indicated by the dashed line. **c** Percentage of the genome with clonal and sub-clonal copy number aberrations (CNAs). **d** Total number of mutations/Mb. **e** Total number of driver gene alterations per sample. Mean of 1.95 shown by solid black line. **f** *TP53* and *ARID1A* mutations. **g** Frequency of whole-genome doubling (WGD) and estimated biopsy cellularity calculated by Battenberg. **h**–**j** Three cases highlighted as outliers in the continuum. **h**, **i** Examples of dysplastic cases lying to the left of the continuum (marked with a yellow asterisk in a black circle in **a**). **j** Indolent case with evidence of chromothripsis (marked with a blue asterisk in a black circle in **a**). For each case, the clinical details of age, gender, maximum length of the BE segment (cm), smoking status, grade of the patient, total follow-up length and computational cellularity of the biopsy are given. Genomic statistics and mutations in driver genes are detailed at the bottom. Circos plots to the right of each case summarise the dominant SV events occurring in each sample.

It was striking that the SV burden was distributed across the cohort as a continuous variable rather than forming a dichotomy according to whether dysplasia was apparent phenotypically (Fig. 2b and Supplementary Fig. S1d, e). This was also true for the BE biopsies taken from adjacent to EAC which were dispersed across the continuum irrespective of dysplasia grade (Fig. 2a, b). Overall, dysplastic cases tended to have a higher SV burden, but the degree of genomic instability was not always reflected in the histopathological grading.

The ordering according to the increasing SV burden was generally commensurate with other genomic features, including the proportion of the genome affected by CNAs, mutation burden, and driver gene alterations (Fig. 2c–e). In contrast to the other features, alterations in *TP53* (mutation and loss) were only observed in dysplastic samples, affecting 65% of cases (39/60). There was a significant trend towards mutual exclusivity between *TP53* loss and mutations in *ARID1A* (Fisher’s exact test, *p*-value = 0.013), affecting 15% of indolent and 17% of dysplastic samples (Fig. 2f and Supplementary Fig. S2a). Whilst most samples with large numbers of SV events were mutant for *TP53*, this was not always the case: both wild-type *TP53* cases with a high degree of rearrangement and mutant *TP53* cases with few SVs were observed (Fig. 2b, f). There was no significant difference in the sub-clonal to clonal proportions of CNAs in *TP53* wild-type vs. mutant samples. Chromothripsis was seen in two cases which were wild-type for *TP53:* both non-dysplastic BE samples taken adjacent to EAC. Early loss of the tumour suppressor gene *CDKN2A* was exhibited in 52% (14/27) of indolent cases. Other driver events occurred at low frequency, mainly in dysplastic cases (Supplementary Fig. S2a). Whole-genome doubling (WGD) was observed in 18% (11/60) of dysplastic samples compared with 4% (2/46) BE sampled at the cancer time point. These two cases of BE adjacent to cancer were dysplastic (Fig. 2g). Notably, none of the indolent cases exhibited WGD. Hence, *TP53* and WGD appear to be tightly associated with dysplasia and invasive cancer suggesting they occur late in tumour development. The accumulation of SVs appears to be a gradual process throughout the disease natural history. To elucidate this further we evaluated the ordering of events taking advantage of the clonality information available from the high depth WGS.

**Fig. 3:**
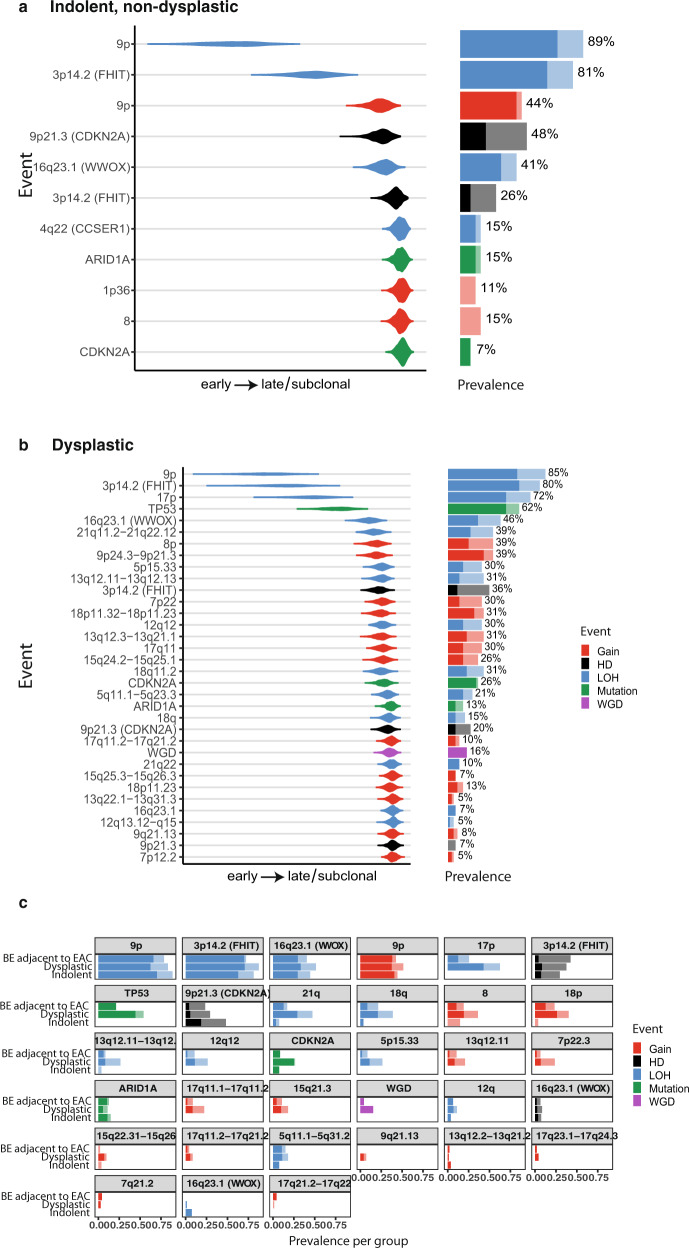
Timing of genomic events. The timing of specific genomic events for indolent (**a**) and dysplastic (**b**) samples, showing the relative ordering of copy number events, driver mutations and WGD. **c** Prevalence of copy number events, driver mutations and WGD per group (indolent, progressed, BE adjacent to the tumour). The events are ordered in the relative order of occurrence determined by the Plackett-Luce model. The lighter colours indicate sub-clonal events. LOH loss of heterozygosity, HD homozygous deletion, WGD whole-genome doubling.

Our timing analysis, which used a data-driven approach to incorporate all driver events (described in the “Timing of copy number changes and driver mutations” subsection), revealed differences in the evolution of indolent and dysplastic BE samples (Fig. 3). Although the losses of chromosomes 3p and 9p were early (mostly clonal) events in both indolent and dysplastic cases, 17p loss, followed by *TP53* mutation, was observed only in the dysplastic cases. Gains (chromosomes 7,8,13,15,19) and WGD were inferred to occur later in the dysplastic samples and were not recurrent events or present (in the case of WGD) in the indolent group. Overall, as expected there were more gains and losses in the dysplastic cases than the indolent cases, and these changes had occurred after the *TP53* mutation.

We did not see any difference in the fraction of sub-clonal vs clonal CNVs in *TP53* wild-type vs mutant samples (mean proportion of all CNAs sub-clonal 0.55 for wild-type vs 0.56 for mutant, *p*-value = 0.43). We observed more extensive copy number heterogeneity within the dysplastic samples (median proportion of the genome with sub-clonal CNA 10.2% ± 18.3%) in comparison with the indolent group (0.04% ± 3.4%) (*p*-value = 4.091e−07, one-sided Wilcoxon rank-sum test). The dysplastic samples showed a higher level of clonal copy number aberration than the indolent samples (median proportion of the genome with clonal CNA 4.2% ± 26.7% for dysplastic, 0.3% ± 0.04% for indolent samples, *p*-value = 2.644e−06, one-sided Wilcoxon rank-sum test). The disparity for sub-clonal copy number aberrations was even greater (median proportion of the genome with sub-clonal CNA 10.2% ± 18.3% for dysplastic, 0.04% ± 3.4%, *p*-value = 4.091e−07, one-sided Wilcoxon rank-sum test) implying increased heterogeneity as well as increased genomic instability in cases that had progressed to dysplasia.

We next looked at mutational signatures occurring as a result of single base substitution (SBS)^38^ to understand at what point in the progression they develop. In contrast to the highly heterogeneous SV counts, these signatures were more homogeneous across all disease stages, including in non-dysplastic, indolent cases (Supplementary Fig. S1f). In particular, signatures SBS17a/b (T>C/G mutations in tri-nucleotide context), known to be hallmarks of EAC^15,16,36^, were seen in all non-dysplastic and indolent BE suggesting that these mutational processes are laid down at the inception of metaplasia independent of subsequent progression to dysplasia.

We compared the non-dysplastic, indolent cases to a small subset of 12 high-quality, non-dysplastic biopsies sampled from patients in the years (median 42.5 months; range 15–100 months) prior to their progression to dysplasia (termed “pre-progressors”) (Table 1). There were no significant differences in the mutation burden or number of SVs between these groups (Supplementary Fig. S3a, b). Furthermore, we noted that *TP53* mutations were not detected in all but one pre-progressor (Supplementary Fig. S3c). For another pre-progressor, despite the biopsy being non-dysplastic and *TP53* wild-type, we noticed CNAs, predominantly at a sub-clonal level, in multiple chromosomes. The patient went on to progress to HGD only 15 months later. This again highlights that the phenotypic change is a late and subjective manifestation of the molecular alterations. When considering genes affected by the SVs, we observed that *RUNX1* which encodes the runt-related transcription factor was rearranged significantly more in pre-progressor than indolent samples (*p*-value = 0.04, Fisher’s exact test) (Supplementary Fig. S3d). This is notable because it is the only feature that we found to define this pre-progression stage.

**Fig. 4:**
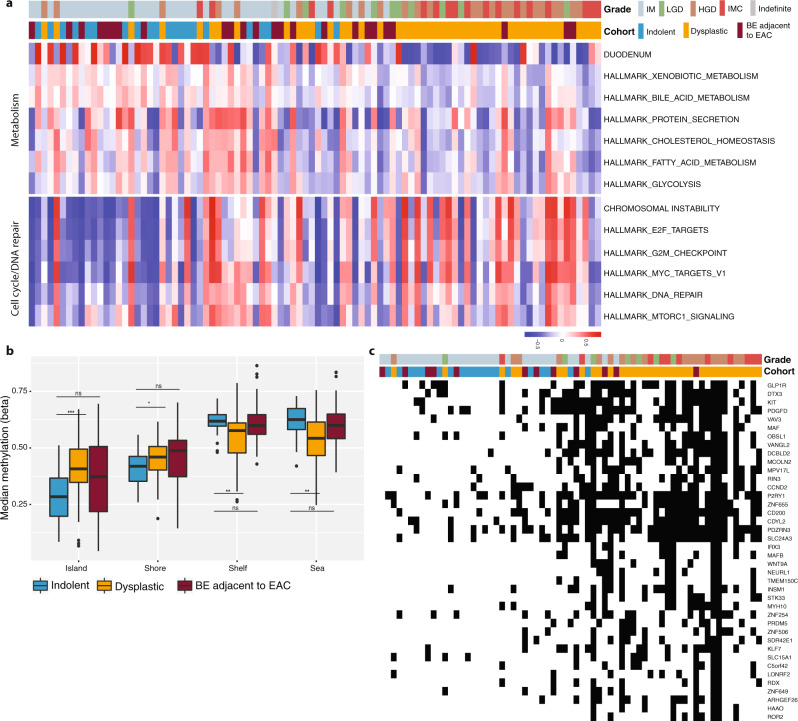
Expression pathway and methylation analysis of the continuum. **a** Gene set enrichment analysis revealed the differential enrichment of genes involved in two main pathways, along the continuum: metabolism and cell cycle/DNA repair. Samples are ordered based on the SV continuum in Fig. 2, with highest patient grade and cohort group denoted at the top. **b** Median methylation levels for the cohort groups within different regions of the genome in relation to the CpG islands: island, shore (up to 2 kb from CpG island), shelf (2–4 kb from CpG island) and sea (>4 kb from CpG island). *N* = 100. Boxplot centre line denotes median, box limits are upper and lower quartiles, whiskers denote 1.5* the interquartile range. Statistical significance was calculated using Wilcoxon signed-ranked test. ns non-significant, *p-value < 0.05, **p-value < 0.5, ***p-value < 0.001. **c** Integrated gene expression and methylation data. Patients are aligned along the same SV continuum order. Thirty-seven genes related to the Wnt signalling and cell cycle pathways for which altered expression followed a gain in promoter methylation and for which there was a significant change in expression with progression.

Methylation and transcriptomic changes were seen alongside the increasing SV burden. For this analysis, we took advantage of a set of hallmark gene signatures representative of immune cell subsets, chromosomal instability and expression in normal tissues^39,40^. Using gene set enrichment analysis, as one progresses from left to right along the SV continuum, there is a clear downregulation of expression patterns related to metabolic pathways, including fatty acid and bile acid metabolism, and a gradual upregulation of signatures related to cell cycle checkpoint, DNA repair and chromosomal instability (CIN) (Fig. 4a). This metabolic downregulation could be in keeping with the loss of the differentiated intestinal metaplasia phenotype which is well-recognised histologically with progression^41,42^. We also identified a third subtype with significant upregulation of inflammatory and immune processes (Supplementary Fig. S4a). This cluster comprised a small number of cases that were mainly from BE sampled adjacent to cancer and may reflect the altered microenvironment surrounding an invasive tumour. Deconvolution of the immune cell types from the expression data revealed significantly higher proportions of T effector memory cells and cytotoxic T cells than in the main, pre-cancer BE cohort (*p*-value < 0.01; Kruskal–Wallis test; Supplementary Fig. S4b).

A quantitative analysis of the degree of methylation (categorised as CpG island (CpGi), shore (<2 kb outside CpGi boundaries), shelf (2–4 kb outside CpGi boundaries), and open sea (the rest of the genome)) showed a significant gain in methylation in CpGi in promoters and a loss in open sea regions with the degree of dysplasia (Fig. 4b). These events can result in the silencing of tumour suppressor genes and genome instability. Integrated analysis of methylation and gene expression status identified 223 genes with altered expression following a gain in promoter methylation. Gene ontology of those silenced genes showed enrichment of transcription regulation machinery (Supplementary Fig. S4c), as well as alterations in genes related to wnt signalling and cell cycle pathways (Supplementary Fig. S4d, e). When these genes were considered per patient and placed on the continuum, they were seen to be significantly more altered with progression (Fig. 4c).

**Fig. 5:**
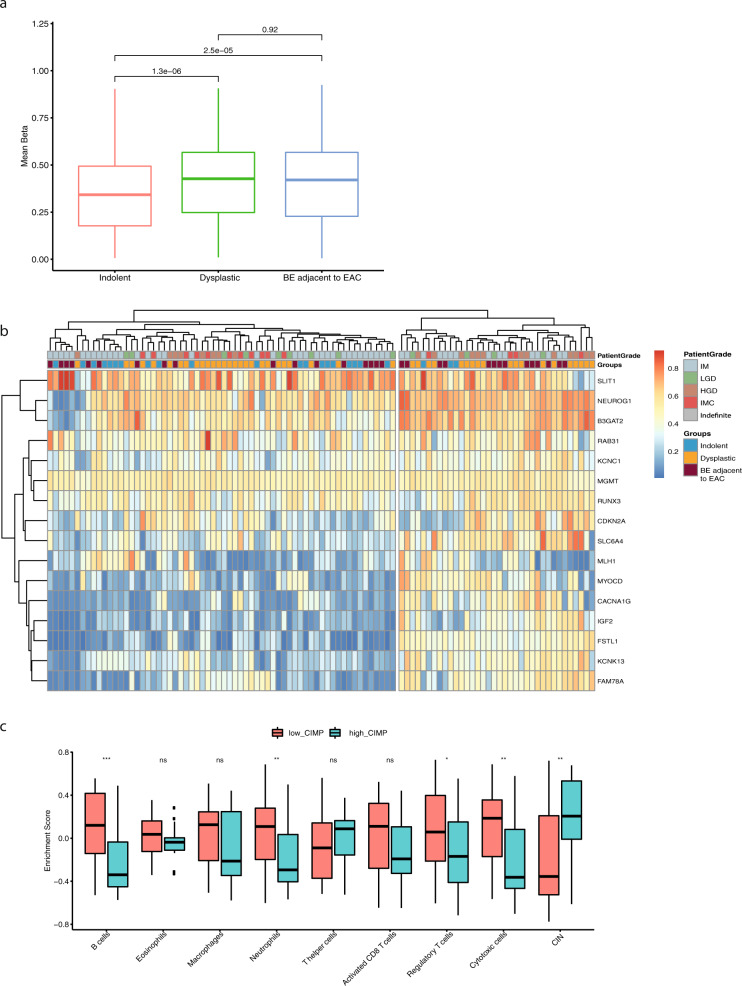
CIMP marker analysis. **a** Boxplot representing mean methylation (beta) of CIMP marker genes across three groups of BE cases. *N* = 100. Boxplot centre line denotes median, box limits are upper and lower quartiles, whiskers denote 1.5* the interquartile range. Statistical significance calculated using Wilcoxon signed-ranked test. **b** Heatmap showing methylation levels for all CIMP marker genes and grouping of samples into low and high CIMP groups. Annotation of samples are shown on the top. **c** Enrichment of different immune cell types and chromosomal instability in low and high CIMP groups. Boxplot centre line denotes median, box limits are upper and lower quartiles, whiskers denote 1.5* the interquartile range. Statistical significance was calculated using Wilcoxon signed-ranked test. ns non-significant, *p-value < 0.05, ***p-value < 0.001.

To determine how methylation events correlate to genomic instability and immune signatures we quantified methylation levels for 16 known markers of CpG island methylator phenotype (CIMP) in gastric and colorectal cancer^43–45^. This showed that compared to indolent cases, dysplastic and Barrett’s adjacent to EAC cases show elevated levels of methylation in CIMP genes (Fig. 5a). When clustered according to the level of CIMP, cases with high CIMP show significantly higher CIN with low immune infiltration when compared to that low CIMP (Fig. 5b, c).

There are lessons that can be learned from outliers so we examined cases with an unexpected SV status when considering their grade of disease. Firstly, we considered the four dysplastic cases furthest to the left of the SV continuum, with few molecular aberrations (marked by yellow asterisks in Fig. 2a; two of which are detailed in Fig. 2h, i). Importantly, all but one (the dysplastic case lying furthest to the left) had reasonable cellularity, suggesting that the analysis was reliable. All were wild-type for *TP53* with no signs of the complex rearrangements or copy number changes evident in other *TP53* wild-type dysplastic cases further right in the continuum. Notably, three of the four patients were female. This is in keeping with our previous driver gene analysis for EAC^13^ in which female patients were more likely to be *TP53* wild-type. Even though these samples were considered as dysplastic at a strict pathology consensus review, at a genomic level they looked stable with very few alterations. This suggests that something else is driving their dysplasia or else perhaps they have been over-diagnosed and would remain indolent. All four patients went on to have treatment for their dysplasia. We also observed one non-dysplastic case (marked by a blue asterisk in Fig. 2a, j) to be an outlier due to early signs of chromothripsis in chromosome 9. Chromothripsis is usually not seen in benign disease, although it has been detected in benign uterine leiomyomas^46^. Interestingly, despite the presence of chromothripsis, the patient has shown no progression after 52 months of follow-up. We did not identify any indolent, non-dysplastic cases to the far right of the continuum suggesting that a degree of genomic instability leads inevitably to dysplasia or cancer. Overall, these analyses suggest that consideration of the genomic landscape and the degree of structural variation adds to the phenotypic assessment of dysplasia.

### Complex patterns of clustered rearrangements occur in dysplastic cases

Given that SVs contributed so strongly to the variance of the cohort, we next undertook a more detailed analysis of the patterns and types of rearrangement. SVs were classified based on the type and size of SV event^47^.

**Fig. 6:**
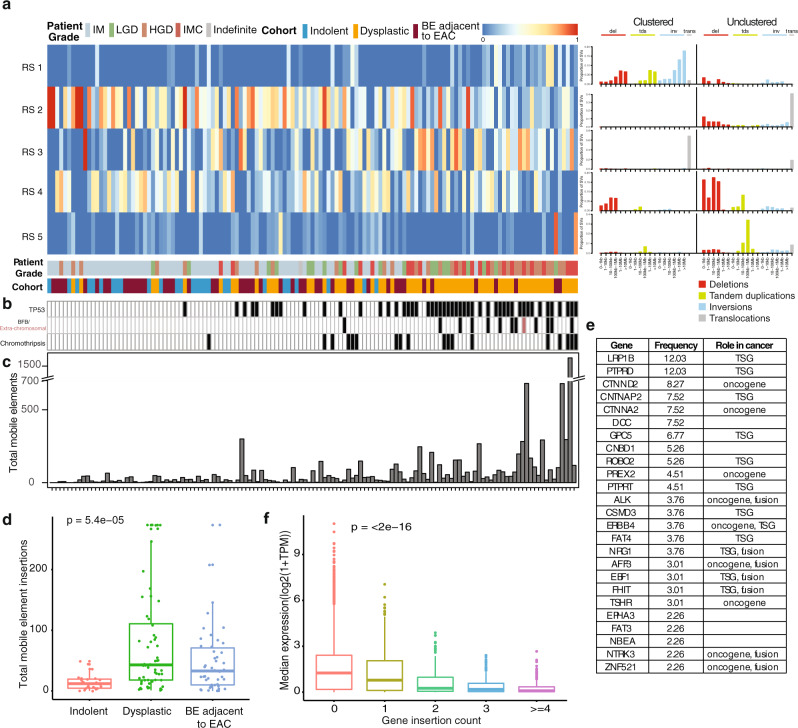
Complex structural variant analysis: mobile element insertions. **a** Structural rearrangement signature (RS) proportions in the cohort: RS1 is dominated by clustered rearrangements; RS2 by unclustered translocations and deletions; RS3 by clustered translocations; RS4 is dominated by deletions; RS5 by tandem duplications >100 kb. Samples remain ordered by the total number of SV events with patient grade and cohort given. **b** *TP53* mutation, breakage-fusion-bridge cycles (BFB) and chromothripsis events in the cohort. **c** The total number of mobile elements in each sample along the continuum. **d** Distribution of total mobile element insertion across different grades of samples. Indolent (*n* = 27), Dysplastic (*n* = 60), BE adjacent to EAC (*n* = 46). Boxplot centre line denotes median, box limits are upper and lower quartiles, whiskers denote 1.5* the interquartile range. Kruskal–Wallis test was used to compare groups. **e** Twenty-eight genes with oncogenic and tumour suppressor roles affected by recurrent (>3) mobile element insertions (as annotated by the Cancer Gene Consensus). **f** All genes are grouped by number of mobile element insertions and then their expression plotted. 0 (*n* = 17564), 1 (*n* = 1156), 2 (*n* = 277), 3 (*n* = 139), ≥4 (*n* = 190). Boxplot centre line denotes median, box limits are upper and lower quartiles, whiskers denote 1.5* the interquartile range. Kruskal–Wallis test was used to compare groups.

**Fig. 7:**
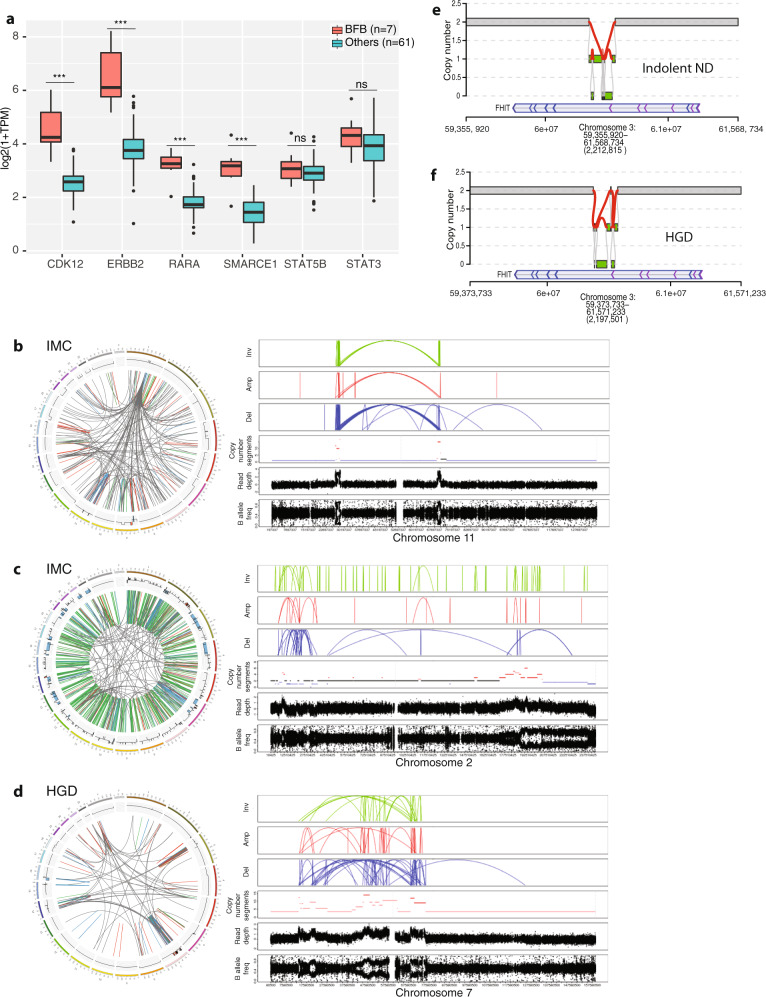
Breakage-fusion-bridge events and case examples of complex rearrangements. **a** Comparison of expression levels of genes both within (*CDK12*, *ERBB2*, *RARA*, *SMARCE1*) and outside (*STAT5B*, *STAT3*) the genomic locus undergoing break-fusion-bridge (BFB) events for samples affected by BFB to those without. BFB cases, *n* = 7, other cases, *n* = 61. Boxplot centre line denotes median, box limits are upper and lower quartiles, whiskers denote 1.5* the interquartile range. ***p-value < 0.001, ns no significance. Statistical significance was calculated using Wilcoxon signed-ranked test. **b**–**d** Circos plots for specific cases as examples of the complex rearrangements seen in dysplastic cases. The outer circle represents each chromosome. Translocations are indicated by the grey lines arcing from one region of the genome to another; the green lines indicate tandem duplications; red indicates deletions; blue signifies inversions. Adjacent to the circos plots are magnified regions showing the clustered events and resultant focal CNAs. **b** An IMC case, with extra-chromosomal DNA and extremely high copy number in two arms of chromosome 11, whereby one locus contains the *CCND1* driver gene. **c** An IMC case dominated by tandem duplications and also showing characteristics of sub-telomeric BFB and chromothripsis in chromosome 2. **d** A HGD case displaying evidence of chromoplexy in chromosomes 3, 7, 16, 18 and X, as well as a complex BFB event in chromosome 7, involving the driver gene *EGFR*. **e**, **f** Two cases, one indolent, non-dysplastic and one with HGD, both displaying a distinct pattern of deletion in an area of low copy number junctions termed “rigma” at the fragile site *FHIT* locus.

Using non-negative matrix factorisation to perform a de novo analysis we identified five rearrangement signatures. These were numbered as previously described^48^, but it should be noted that RS1 was not described in the previous breast cancer cohort analysis (RS1 to RS5, Fig. 6a). Signature RS1, characterised by clustered rearrangements and RS5, marked by tandem duplications (>100 kb), were enriched mainly in cases from the dysplastic group. RS1 can result from an aggregate of multiple complex events including chromothripsis (13.4%, 18/134 of which 11 were in dysplastic cases) and breakage-fusion-bridge cycle (BFB) (9%, 12/134 of which 11 were in dysplastic cases) (Fig. 6b). Of note, in more than 50% of these cases, the BFBs were observed in chromosome 17, which harbours the oncogenes *ERBB2* and *CDK12*, (Fig. 7a and Supplementary Fig. S5), for which expression was significantly higher compared to other genes outside the locus such as *STAT5B*, *STAT3* (Fig. 7a). Such events surrounding *ERBB2* have been previously observed in breast cancer^49–51^. One IMC case had clustered inversions with extremely high copy number (>10 copies) in both arms of chromosome 11, characteristic of an extra-chromosomal event, with amplification of the locus containing the *CCND1* driver gene, resulting in an elevated expression (log2(1+TPM)) 5.25 greater than the median expression from cases with normal copy number (Fig. 7b and Supplementary Fig. S6a).

In many cases, there were multiple complex events. For example, an IMC case was dominated by tandem duplications and also showed characteristics of sub-telomeric BFB and chromothripsis in chromosome 2 (Fig. 7c and Supplementary Fig. S6b). Similarly, another HGD case displayed evidence of chromoplexy in chromosomes 3, 7, 16, 18 and X (Fig. 7d), as well as a complex BFB event in chromosome 7, involving the driver gene *EGFR* (Supplementary Fig. S6c). In a recent pan-cancer study^52^, new complex events have been characterised that were also identified in our cohort. A phenomenon they termed rigma, which contains distinct patterns of deletions in areas of low copy number junctions, was prevalent across all grades, occurring in 78% (114/146) cases, particularly at fragile sites (mainly *FHIT* (Fig. 7e, f and Supplementary Fig. S6d), *WWOX*) and, in some instances, at the *CDKN2A/B* locus. In contrast, a phenomenon named pyrgo, with a characteristic pattern of amplification in areas of low copy number junctions, was seen in only 3% of cases (Supplementary Fig. S6e, f).

Signature RS3, characterised by clustered translocations, showed a strong association (*r* −0.5, *p*-value < 0.0001) with LINE-1 (L1) retrotransposon activity (Fig. 6a, c). As noted earlier, mobile element insertions (MEI) are known to be prominent in EAC^18^ but it has not been clear at which stage in the progression they occur. We identified them in 96% of BE samples ranging from 1 to 1700 insertions per sample. We observed that MEI activity is generally low in the early stages of BE and increases significantly during progression through the stages of dysplasia (Fig. 6d). MEIs were very diverse in BE sampled from adjacent to EAC (Fig. 6d and Supplementary Fig. S3e). In most cases, the source of these MEI could be mapped to known loci: *TTC28*, *SNX30*, *PHACTR1* and several non-coding region loci mapping to chrX(11725366-11731400), chr14(59220385-59220402), chr1(119394975-119401004), chr6(29920213-29920223) and chr15(85140894-85140909) (Supplementary Data File 1). In total, recurrent (>3) MEI were observed in 329 protein-coding genes of which 28 genes are annotated in the Cancer Gene Consensus with either oncogenic (ALK1, ERBB4, TSHR, PREX2, CTNNA2, CTNND2) or tumour suppression roles (EBF1, LRP1B, PTPRD, GPC5, ROBO2, PTPRT) (Fig. 6e). Considering expression, we observed that genes with increased MEI tended to show lower expression, consistent with PCAWG studies^18,53^ (Fig. 6f). The relative proportion of other signatures, RS2 (dominated by unclustered translocations), and RS4 (dominated by deletions), negatively correlated with the number of SVs.

When considering genes directly affected by SVs, we observed that some recurrent fragile sites (*FHIT*, *WWOX*) were equally affected across all disease grades while others (*DMD*, *PDE4D*, *MACROD2*, *PARD3B*) were significantly altered in dysplastic compared with indolent cases (Supplementary Fig. S6d). In terms of protein-coding genes, the *INK4/ARF* locus was rearranged significantly more in indolent cases than in either pre-progressor (Fisher’s exact test, *p*-value = 0.03) or dysplastic cases (Fisher’s exact test, *p*-value = 0.01). Other protein-coding genes such as *ERBB2, EGFR, MYC* were affected in very small numbers of cases but did not significantly differentiate dysplastic cases, except for *CDK14* (Fisher’s exact test, *p*-value = 0.02).

Hence, cases with a high SV burden also tend to display increased retrotransposon activity and complex rearrangements, including a high prevalence of events that are associated with genome instability such as chromothripsis and BFB events in chromosomes 9p and 17q.

## Discussion

This multi-omic analysis of a large cross-sectional BE cohort with very detailed clinical annotation, representing all pre-cancer disease grades from non-dysplastic to dysplasia and intramucosal cancer, demonstrates a highly heterogeneous genomic landscape between patients, even when their histological phenotype is similar. Rather than the CNA or mutation burden, the SV landscape best describes the genomic variability across the cohort and, in keeping with this, there is an increasing number and complexity of rearrangements and LINE-1 retrotransposon events with advancing grade. Whilst these complex, large-scale rearrangements and retrotransposons are increasingly understood to have roles in multiple cancers, it has not been appreciated to this extent in pre-malignant disease, although LINE-1 retrotransposon activity has been demonstrated previously in very small numbers of pre-cancers, namely including benign BE^54^ and colorectal adenomas^55,56^.

The types of structural rearrangements are varied and involve a range of gene loci. Hence, there is no single gene-specific molecular feature defining the clinically-assigned stepwise, phenotypic, pathological grades apart from an enrichment of SVs affecting *RUNX1* in pre-progressors. Mobile element insertions are seen in early stages with a very low prevalence but increase significantly with the number of SVs observed. This increased activity might be due to hypomethylation of repressed heterochromatin regions harbouring LINE-1 elements causing chromatin to be more accessible and thereby triggering active expression of such elements which are then integrated into other parts of the genome. Hypomethylation is commonly observed in dysplastic BE (Fig. 4b). Here, we examined methylation from CpG probes sparsely distributed across the genome which does not provide a complete picture of heterochromatin with repetitive elements. Further insight could be achieved with whole-genome bisulfite sequencing or Tet-assisted pyridine borane sequencing (TAPS)^57^.

Fragile sites are recurrently altered early, from the indolent stage. We observed whole-genome doubling to be a relatively late event, and present only in dysplastic samples. Along with these genomic alterations, both methylation and transcriptomic data suggest global changes in the epigenetic landscape with early signs of DNA damage, aberrant cell cycle pathway and chromosomal instability in dysplastic cases.

In multiple previous studies, BE sampled at the cancer time point has been used to infer the biology of disease progression^20–22,25^. Caution should then be exercised when extrapolating findings from analysis of BE samples taken adjacent to EAC, since there may be a field effect on the tissue from which the cancer arises^58–60^, and our data suggest that the genomic alterations are not always in keeping with the grade of dysplasia. In our cohort comparison of pre-progressors with indolent cases, there were no significant differences in the mutation burden, CNAs or number of SVs between these groups. Whilst initially this appears to contradict previous work by Killcoyne et al.^37^, we used absolute, rather than relative, copy number in this analysis, taking cellularity into consideration. Furthermore, we were unable to look at low-level CNAs (<3) and to compare pre-progressors with progressors due to the smaller sample size.

The significant strength of this study lies in the meticulous curation of a large cohort of cases with whole-genome sequencing data. The triple, independent consultant pathologist reviews provided confidence in the pathology of the sequenced biopsies. Patients had long-term follow-up and clear records of any treatment intervention. We collated detailed, complete clinical information on each case. This makes this cohort invaluable for the future integration of this information into progression models. The limitations include the single biopsy per case from one time point. We are thus conducting analyses across multiple time points and levels of the BE segment to determine the within patient heterogeneity in more detail but unfortunately for this cohort, the material available with the temporal and spatial resolution is limited. Given the limited sample sizes available for emerging sequencing datasets in BE^61,62^, it will be important to combine the data to provide the necessary power to discover additional driver events for progression.

**Fig. 8:**
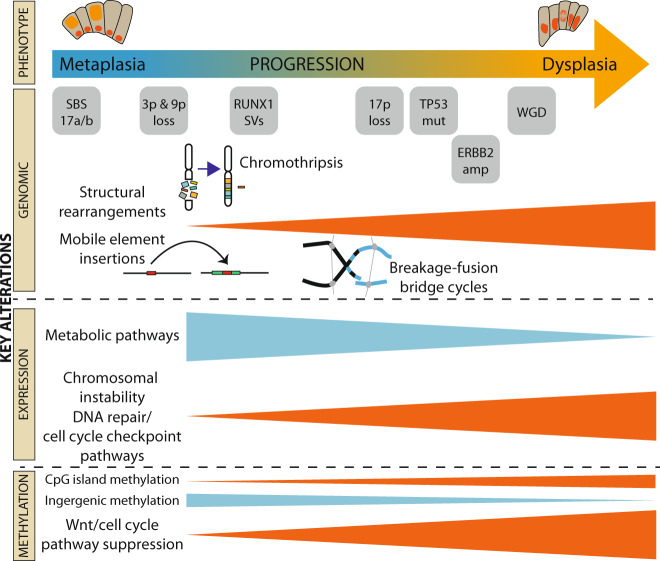
Summary of main events. Overview of the main events across the different sequencing modalities, seen to be associated with progression from the indolent, non-dysplastic to the dysplastic state.

In summary, we have shown here that there is a wide spectrum of genomic events between patients with the pre-malignant condition BE. These include complex structural rearrangements and mobile element insertions which can occur early in the natural history of cancer progression. This is notable since these alterations have generally previously been associated with advanced epithelial cancers. The phenotypic appearances of the pre-malignant epithelium can thus belie the degree of genomic instability and underline the limitations of using a subjective assessment of the cellular phenotype in assessing the propensity for invasion. This continuum is defined by an increasingly complex landscape of structural rearrangements and mobile element insertions, often but not always preceded by *TP53* mutation. We infer from these observations that molecular features accumulate over time until the resulting genomic instability begins to increase exponentially and tips the balance to a “free-fall” towards progression (Fig. 8). Incorporating a more quantitative assessment of the molecular alterations and tracking them over time could facilitate patient management decisions, especially in cases with indefinite pathology or lack of pathologist concordance. Efforts to better understand the triggers for chromosomal breakages and rearrangements that underlie progression to cancer will be important for improving clinical prevention strategies.

## Methods

### Ethics

All Barrett’s esophagus (BE) patients were selected from our Cell Determinants Biomarker (REC no. 01/149), BEST2 (REC no. 10/H0308/71) and OCCAMS (REC. no. 10-H0305-1) trials. Cell Determinants Biomarker is an observational trial that focuses on determining biomarkers to identify patients with BE who have a higher risk of progression to esophageal cancer. BEST2 is a case:control observational study using the CytospongeTM to test for diagnosing BE. OCCAMS is an observational study to determine the molecular drivers of EAC. Ethical approval for these trials was from the East of England-Cambridge Central Research Ethics Committee. Tissue was obtained with written, informed patient consent. All relevant ethical regulations were correctly followed and samples were fully anonymized.

### Cohort creation

Patients undergoing surveillance at Cambridge University Hospitals NHS Trust are consented prospectively to a biomarker and genomic characterisation study. Patients did not receive any compensation for taking part in these studies. The cohort was constructed retrospectively from this bioresource, from all three studies, using strict criteria as described below. From these patients, one research biopsy had been taken every 2 cm of the BE segment at endoscopy. These biopsies were snap-frozen in liquid nitrogen and stored at −80 °C. The biopsies were taken alongside the diagnostic biopsies which were fixed in formalin and embedded in paraffin (FFPE). Histology reports of these FFPE diagnostic biopsies were used to identify the potential patients with the different grades of disease. Patients were excluded if they had progressed past the grade of interest, either before or at a later date. This gave certainty of the grade being sequenced and negated the risk of local effects from prior higher grades. Samples with an imminent future higher grade of progression were excluded because of the possibility that the higher grade was already present and missed at BE surveillance. Patients were also excluded if they had received previous ablative treatment of their BE. Biopsies representing the independent grades could not be adjacent to cancer. Non-progressing patients with long follow-up and long segments were selected where possible and pre-progression samples were taken as far in advance of progression as available. In addition, cases of BE adjacent to cancer were selected as a comparison. These frozen samples were taken either from the esophagectomy specimen or at the staging endoscopy and BE was sampled at the greatest distance possible from the tumour to avoid potential tumour contamination.

Strict selection criteria were implemented to ensure that only the highest cellularity biopsies, with the agreement of histological grade were sequenced. Potential biopsies were placed into OCT and a single section cut and stained with haematoxylin and eosin (H&E). These were reviewed by a consultant pathologist to assess the composition of the biopsy. For the pre-cancer BE cohort, any potentially suitable biopsies were then reviewed independently by a further 2 consultant pathologists. All pathologists were blinded to the grade of the patient. Sample grade was determined by an agreement of at least two pathologists. Samples with no agreement were reviewed by the 3 pathologists together to reach a consensus. Dysplastic samples for sequencing had to have a pathological cellularity of dysplasia of >30% and were included even if the dysplasia was not all the highest grade the patient was known to have. Non-dysplastic BE biopsies had to contain intestinal metaplasia (IM). Samples with only gastric metaplasia (GM) were excluded because this phenotype of BE has an extremely low risk of progression and surveillance is not recommended for short-segment GM^63^. For the BE adjacent to cancer, H&Es were reviewed independently by two pathologists and only dysplastic cases were reviewed by a third. 23 of genomes were previously published^22^, but not the expression or methylation for those same patients. Biopsies with any tumour contamination were excluded. Across the whole cohort, no squamous epithelium could be present in any sample; however, samples with inflammation were not excluded. Duodenum was used as the germline reference as blood had generally not been collected. Where not available, blood or normal squamous esophagus (verified with H&E staining) were used in that order.

In total, 1161 frozen biopsies from 315 patients were cut and reviewed for inclusion (Fig. 1b). Information about the final sample cohort for inclusion (study, sample type, patient grade) are included in Supplementary Date File 2.

### DNA/RNA extraction

Whole frozen tissue biopsies were homogenised on the Precellys® and DNA and RNA were extracted using the AllPrep DNA/RNA Mini Kit (Cat No. 80204; Qiagen®, Germany), as per protocol and all additional optional steps were performed to maximise yield. DNA was eluted in 100 μl EB buffer and RNA in 30 μl RNA-free water. RNA was initially quantified using the Nanodrop. DNA was quantified using the Qubit® Low Sensitivity assay on the Qubit® 2.0 fluorometer (Invitrogen, Life Technologies, UK). 20 ng/μl concentration of DNA was required for whole-genome sequencing. Blood for germline reference was extracted using the QIAmp Blood Maxi kit (Cat No. 51192; Qiagen®, Germany).

### DNA library preparation and sequencing

A total of 109 pre-cancer BE biopsies (matched BE-germline) and 47 BE adjacent to cancer from 156 patients were sent for sequencing, under Illumina contracts. 100-bp paired-end sequencing was carried out to an average depth of 50× for BE and 30× for matched normal. Ten cases were subsequently excluded: six cases were deemed to have too low a concentration for sequencing during Illumina QC; two samples were found to be mismatched with their normal tissue; one had very few mutations called due to poor coverage and low cellularity, and one was removed by us as a further Pathological review deemed it to have no dysplasia. The final analysed cohort consisted of 146 patients (Supplementary Data File 2).

### Pipelines for variant-calling and genomic events (copy number alterations and structural variants)

The FastQC package 0.11.7 was used to assess the quality-score distribution of the sequencing reads and perform trimming if necessary. Read sequences were mapped to the human reference genome (GRCh37) using Burrows–Wheeler alignment (BWA-mem) 0.7.17^64^. Duplicates were marked and discarded using Picard 2.9.5 (http://broadinstitute.github.io/picard/). Three samples were excluded from the sequenced cohort: two were mismatched to their germline sequences and one had passed QC but had inadequate coverage so that Strelka was unable to call mutations (Fig. 1). No samples had evidence of microsatellite instability using MSIsensor^65^. Overall, 98% of the known genome was sequenced to at least 10× coverage and 60% to a 50× coverage. The whole cohort had at least 85% aligned bases within a read with a Phred quality of 20 or higher and with a base Phred quality of at least 20. Somatic mutations and indels were called using Strelka 2.0.15^66^ with additional filters as previously published^13,67^.

Structural variants were identified using Manta 0.27.2^68^. Discordant reads and split reads were used to identify putative breakpoint junctions. These methods have been compared to other variant callers in the ICGC benchmarking exercise and have among the best sensitivity and specificity^69^. Single nucleotide polymorphisms were called using GATK HaplotypeCaller 3.2-2^70^. Allele count analysis was performed using GATK ReadCountWalker 0.2.2 at SNP positions from the 1000 Genomes Project, and at single nucleotide variant positions.

Absolute copy number was called using Battenberg 2.3.2^71^ which was able to call sub-clonal copy number for the clonality analysis.

Samples were considered as whole-genome doubled (WGD) if their ploidy estimate was greater than 2.6 in Battenberg.

### Driver gene identification

For identifying genes recurrently altered in BE, we used MutSigCV and dndscv for identifying genes based on variants. We used GISTIC 2.0, for identifying genes undergoing CN alterations. Along with these genes, in our previous work in EAC^13^, we were able to identify other genes which play a vital role in the development of EAC and might be altered in the late stage of progression, in order to understand their prevalence in BE, we also considered those too.

### Mutational signature analysis

A de novo discovery of mutational signatures was performed using the non-negative matrix factorisation (NMF) methodology described by Alexandrov et al.^72^ using the Python version of SigProfiler 2.5.1.8 (https://www.mathworks.com/matlabcentral/fileexchange/38724-sigprofiler). For identifying optimal de novo signatures, NMF was run for 2–10 ranks for 1000 iterations. This process identified 8 optimal signatures which, when decomposed, mapped to 14 known signatures. The de novo signatures were compared to the 50 known published COSMIC signatures (https://cancer.sanger.ac.uk/cosmic/signatures/SBS/).

### Structural variant analysis

Structural rearrangement signatures were identified based on a framework as explained by Nik-Zainal et al.^47^. SVs were classified into 38 categories based on the type and size of SV event, then were further classified into clustered and non-clustered. Events were considered to be clustered in a sample if a region of the genome (1 Mb) was covered by >10 breakpoints. NMF was then applied to these events using Palimpsest 1.0^73^, identifying five optimal signatures.

### Complex events

Both low and high confidence chromothripsis events were identified, based on oscillating copy number events in regions with clustered breakpoints, across all samples using ShatterSheek^74^. Junction Balance Analysis (JabBA) genome graph-based programme^75^ was used for identifying breakage-fusion-bridge cycles (BFBC), extra-chromosomal, pygro, rigma, tyfonas and chromoplexy events. Rigma and pygro were identified as clusters of at least 2 overlapping DEL/DUP-like junctions with JCN less than ploidy and sizes between 10 kbp and 10 Mbp. For mapping BFBC, extra-chromosomal and tyfons defined set of properties were used upon which classification model was trained using pan-cancer data and constraints derived from such model were then used for nominating amplicon into each specified category. As explained, in JabBA, BFBC, extra-chromosomal and tyfonas were identified based on amplicon graph features maximum segmental/interval copy number, the sum of all fold-back inversion JCN divided by maximal interval copy number, the ratio between maximum JCN and maximum interval copy number, and the number of junctions with elevated JCN (thresholded on JCN > 3). A classifier was built on such features, which resulted in a decision tree and arrived at a fold-back JCN R 0.5 to distinguish tyfonas/BFBC amplicons from the double minute/Other amplicon categories. Out of the amplicons with fold-back JCN R 0.5, those with a total number of junctions with high JCN (# R 26) were called tyfonas and the rest were called BFBCs. Within amplicons containing fold-back JCN < 0.5, those amplicons with R 31 high copy junctions distinguished otherwise unspecified amplicons (Other) from regular double minutes, respectively. Whereas chromoplexy was identified from a pool of low-JCN (%3) edge clusters in which junction breakends were no further than 10 kbp away from the next junction breakend. Edge clusters that contained at least three long-range junctions, and whose footprints that occupied at least three discontiguous genomic territories separated by >10 Mbp on the reference, were called as chomoplexy events.

Somatic retrotransposition insertions across all samples were identified using TraFiC^76^ under default settings. We used the annotations as provided by TraFiC for classifying MEI to be germline/somatic and for computing recurrence events hitting to protein-coding genes.

### RNA library preparation

RNA was quantified using the Qubit High Sensitivity RNA kit (Thermo Fisher) and checked for quality (RNA integrity number; RIN) on the Agilent 2100 Bioanalyzer® (Agilent Technologies, USA) using the RNA 6000 Nano kit. Samples with insufficient material, or an incalculable RIN were excluded. There was no other lower limit for RIN inclusion.

Samples were randomised to 3 batches, ensuring an equal spread of RIN values across the batches. Libraries were prepared with an input of 150 ng RNA using the TruSeq Stranded Total RNA High Sensitivity protocol with ribosomal depletion. Samples with less than the specified input, but with >100 ng total were included and this was noted for the analysis. Library quality and quantity were checked using the Agilent 2100 Bioanalyzer with the DNA 1000 kit and KAPA quantification (KAPA Biosystems, Roche, Switzerland) and were pooled according to the Illumina protocol. Samples were run on the HiSeq 4000 instrument to generate 75 bp paired-end reads, aiming for 50 M reads per sample. A mixture of normal expression controls was run on each plate: squamous esophagus, gastric cardia, duodenum. Duodenum mimics the intestinal appearance of BE and it is hypothesised that BE arises from gastric cells. Squamous esophagus is a less useful comparison because it shares few features with the glandular epithelium of BE.

### Pipelines for RNA

RNA sequencing data were trimmed for poor quality bases using Trim Galore (https://www.bioinformatics.babraham.ac.uk/projects/trim_galore/) and was then aligned using STAR^77^ using the ENSEMBL gene annotation. Reads per gene were quantified using the summariseOverlaps function from the GenomicRanges package, which was also later used for computing Transcripts per million (TPM). Normalised expression data were corrected for batch effect using the ComBat function in the sva package 3.20.0^78^. Expression was calculated as log2(1 + TPM). NMF was applied on the top 3000 most variable genes for identifying distinct subtypes. EdgeR^79,80^ was used to compare the differential expression between the different groups using the raw counts. Genes were then ranked on the basis of the fold change of expression and level of significance, which was then used for identifying differentially regulated gene signatures using Gene Set Enrichment Analysis.

### Immune signatures and chromosomal instability

Markers for both immune cell types and chromosomal instability were retrieved from publication^39,40^ and Gene Set Variation Analysis (GSVA) was used to assign enrichment scores to samples based on the expression of different markers in the bulk RNA-seq^81^.

### Copy number driver gene discovery

GISTIC 2.0^82^ was used to identify recurrently amplified and deleted regions from the raw copy number calls; a method that has been previously used in the lab^13^. Peaks were widened by 1 million base pairs up- and downstream and all genes falling within these regions were considered. The expression of each gene from the RNA-seq data was compared for high vs. normal CN samples for each gene and mean expression levels were compared using the Wilcoxon rank-sum test. The Benjamini–Hochberg method was used to correct for multiple testing.

### Timing of copy number changes and driver mutations

To determine the temporal ordering of mutational drivers (*TP53*, *CDKN2A*, *ARID1A*) and copy number aberrations (CNAs) in the cohort, a model based on the Plackett–Luce framework^83,84^ for ordering partial rankings were developed. Copy number information and cancer cell fraction estimates from Battenberg and DPClust^71^ were used to assign the clonality of CNAs and mutations, respectively.

The recurrently aberrant regions in the cohort were identified by performing 1000 simulations, where the copy number aberrations detected in each sample were randomly placed on the genome. The process was repeated for gains, LOH and homozygous deletions. The null hypothesis that a region was not enriched was tested by comparing the number of times an event had occurred in the cohort and the expected number of times the event occurring by chance in the 1000 simulations. The *p*-values were corrected for multiple hypothesis testing using False Discovery Rate, and the enriched segments were identified as those with FDR-corrected *p*-value < 0.05.

For each patient, a possible phylogenetic tree was sampled using the cancer cell fraction estimates derived with Battenberg for the CNAs and DPClust for the driver mutations, and from that, a possible order of the events that had occurred in this patient was obtained. If there was a clonal and sub-clonal gain or loss at the same locus, only the clonal (i.e. the initial) gain or loss was ordered. In addition, the homozygous deletions were assumed to have occurred after a loss of heterozygosity at the same locus. The timing of the CNAs with respect to WGD was determined using the parsimony principle: for losses, if the minor allele copy number was 0, then the loss had arisen before WGD; for gains, if the major copy number was twice or greater than the ploidy, then the gain had occurred before WGD. The timing of the driver mutations with respect to WGD was determined using the number of chromosomes carrying the mutation. If the mutation was on more than one copy, then it had happened before WGD. The enriched events that were not observed in the patient at the time of sampling were assumed to have occurred at a later time point.

Using the Plackett-Luce model implemented in PLMIX 2.1.^85^, these orderings for all patients were combined to arrive at a global value quantifying how early/late each event had occurred in this population. This procedure was repeated 1000 times to get a distribution of the global values for each event.

### Methylation

Methylation profiles for all samples were generated using the remaining extracted DNA on the EPIC 850k array platform (Illumina, US) with 150 ng input. Samples were processed as previously described^67^. All raw data were processed using minfi^86^ and samples with less than 96% capture efficiency were not considered in the analysis. Probes were filtered if they were not significantly detected from background, or were not in the CpG context, had known SNPs in the surrounding locus, aligned to multiple locations in the genome or if they mapped to X and Y chromosomes. Processed methylation data were further normalised using the BETA mixture model BMIQ^87^ implemented in the ChAMP package^88^. Processed data were then corrected for batch effects using *limma*^89^.

### Identifying epigenetically silenced genes

For assessing which genes had undergone transcriptional repression under the influence of gaining methylation in promoter regions, we performed integrative methylation and RNA-seq analysis. For this analysis, we considered samples for which both RNA-seq and methylation were available. For each gene, we identified all probes located 1500 bp both up and downstream from the transcription start site (TSS). We selectively removed all CpG sites that were methylated in normal tissues (mean *β*-value > 0.2). Methylation data were then dichotomised using *β*-value of ≥0.3 as a threshold (as used in TCGA studies^90,91^) for positive DNA methylation, and discarded CpG sites methylated in fewer than 10% of samples. For each probe/gene pair, we then performed the following: (1) categorised samples as either methylated (*β* ≥ 0.3) or unmethylated (*β* < 0.3); (2) compared expression in the methylated and unmethylated groups using the Mann–Whitney test; (3) computed the correlation between methylation beta and expression TPM. We labelled each individual sample as epigenetically silenced for a specific probe/gene pair selected above if for the probes there was a difference in beta (>0.2) between two groups, a difference in the distribution of expression of (adjusted *p*-value < 0.05) and a negative correlation between methylation and expression (*r* < −0.1, adjusted *p*-value < 0.05). Only genes with multiple probes were considered for this analysis and a sample was considered as epigenetically silenced if more than thirty per cent of probes for the corresponding gene were also labelled as epigenetically silenced.

### Reporting summary

Further information on research design is available in the Nature Research Reporting Summary linked to this article.

## Supplementary information

Supplementary Information

Legends Supp figures and data

Supplementary Figure 1

Supplementary Figure 2

Supplementary Figure 3

Supplementary Figure 4

Supplementary Figure 5

Supplementary Figure 6

Supp Data File 1

Supp Data File 2

Reporting Summary

## Data Availability

The sequencing reads for this study have been deposited in BAM format at the European Genome-phenome Archive (EGA) under the following datasets: https://ega-archive.org/datasets/EGAD00001006349 (WGS) and https://ega-archive.org/datasets/EGAD00001006353 (RNAseq). Reads that are not used in the alignment are included to enable any reprocessing. The methylation array data has been deposited in IDAT format: https://ega-archive.org/datasets/EGAD00010001972. These are controlled access data; details on how to apply for access are available on the linked pages..

## Code availability

Scripts for data processing and for generating figures are available upon request by emailing the corresponding author.
