# Supplementary material for: Multi-omic cross-sectional cohort study of pre-malignant Barrett’s esophagus reveals early structural variation and retrotransposon activity
Source: Nat Commun. 2022 Mar 17;13:1407. doi: 10.1038/s41467-022-28237-4 (PMC8931005; doi:10.1038/s41467-022-28237-4)
Supplement: Supplementary file 2 — Legends Supp figures and data [file 41467_2022_28237_MOESM2_ESM.docx]

**Supplementary figure legends**

**Figure S1**

**a, b.** Principal component analysis using genomic variables showing the relative contribution of each variable to the 1^st^ and 2nd dimensions. **c.** Patient order when translocations, and therefore retrotransposon activity, included in the total SV count for the continuum.. **d.** SV counts by pathological grade of patient. IM group includes both indolent and pre-progressor cases. Significance indicated by p-values above plots. IM (n=77), LGD (n=19), HGD (n=28), IMC (n=21). Boxplot center line denotes median, box limits are upper and lower quartiles, whiskers denote 1.5* the interquartile range. Statistical significance calculated using Wilcoxon signed-ranked test. ns = non-significant. **e.** SV counts by patient cohort group. Indolent (n=27), Pre_Progressor (n=12), Dysplastic (n=60), BE adjacent to EAC (n=47).

Boxplot center line denotes median, box limits are upper and lower quartiles, whiskers denote 1.5* the interquartile range. Statistical significance calculated using Wilcoxon signed-ranked test. ns = non-significant. **f.** Single base substitution signature proportions of samples ordered along the SV continuum. n=134.

**Figure S2**

Driver gene alterations across the cohort. Samples ordered by SV count within the continuum. Percentages of samples with each gene mutated given on the left.

**Figure S3**

**a.** Comparison of mutation burden between non-dysplastic (ND) indolent cases (n=27), ND patients prior to progressing to dysplasia (pre-progressor, n=12) and prevalent BE adjacent to EAC (n=47). Boxplot center line denotes median, box limits are upper and lower quartiles, whiskers denote 1.5* the interquartile range. Statistical significance calculated using Wilcoxon signed-ranked test. ns = non-significant. **b.** Comparison of total numbers of structural variants (SV) per sample in ND indolent cases (n=27) and ND biopsies from patients prior to progressing to dysplasia (n=12). Boxplot center line denotes median, box limits are upper and lower quartiles, whiskers denote 1.5* the interquartile range. Statistical significance calculated using Wilcoxon signed-ranked test. ns = non-significant. **c.** Driver gene alterations in ND indolent cases and pre-progressor cases. Percentages of samples with each gene mutated given on the left. **d.** Percentages of each cohort group with SVs affecting each of 17 genes found to have differences between the groups. Asterisks indicate significant differences (p-value < 0.05, Fisher’s exact test). *RUNX1* is significantly more frequently rearranged in pre-progressing than indolent cases. *CDKN2A*/B is more frequently rearranged in indolent than pre-progressing cases. *CDK14* is differentially rearranged in dysplastic and prevalent BE adjacent to EAC cases. **e.** Comparison of total number of mobile elements seen in each group, with the BE adjacent to cancer samples divided based on their histology: either non-dysplastic (ND) or dysplastic. Boxplot center line denotes median, box limits are upper and lower quartiles, whiskers denote 1.5* the interquartile range. Statistical significance calculated using Wilcoxon signed-ranked test. ns = non-significant, * = p-value < 0.05, ** = p-value < 0.5, *** = p=value < 0.001.

**Figure S4**

**a.** Gene set enrichment analysis with hierarchical clustering highlights three biological processes to be differentially enriched across three subtypes identified through a NMF consensus-based approach. n=92. **b.** Deconvolution of the immune cell types from the expression data with assigned enrichment scores per group. Boxplot center line denotes median, box limits are upper and lower quartiles, whiskers denote 1.5* the interquartile range. Kruskal-Wallis test: ns = non-significant, * = p-value < 0.05, ** = p-value < 0.01, *** = p=value < 0.001 **c-e.** Gene ontology of genes silenced by methylation in the integrated expression and methylation analysis. **c.** Enrichment of transcription regulation machinery. **d, e.** Gene ontology and pathways enriched in genes preferentially methylated only in dysplastic cases.

**Figure S5**

Seven examples of cases where break fusion bridge events were observed in chromosome 17, affecting the expression of genes *ERBB2* and *CDK12*.

**Figure S6**

**a.** Most frequently rearranged fragile sites in the cohort. **b, c.** Regions displaying the pattern termed “pyrgo”: characterized as duplications with low copy number junctions. **d.** High density complex rearrangement pattern with fold-back inversions and duplications in chromosome 7 harbouring driver genes such as *EGFR*. **e.** Case with a chromoplexy event, where rearrangements connect multiple chromosome. **f.** Specialised case with patterns of double minutes, with high copy number at a specific locus and clustered inversions.

**Supplementary Data File 1**

List of genes with frequencies of samples being altered by MEI, along with p-value, q-value and role of the gene in cancer. P-values and q-values were computed by Genomic Random Interval (GRIN) model (PMID: 2384812).

**Supplementary Data File 2**

Summary of the samples used, the types of sample and study from which they originated.
