# Supplementary figures and images for: Multi-omic cross-sectional cohort study of pre-malignant Barrett’s esophagus reveals early structural variation and retrotransposon activity
Source: Nat Commun. 2022 Mar 17;13:1407. doi: 10.1038/s41467-022-28237-4 (PMC8931005; doi:10.1038/s41467-022-28237-4)

Figure S1

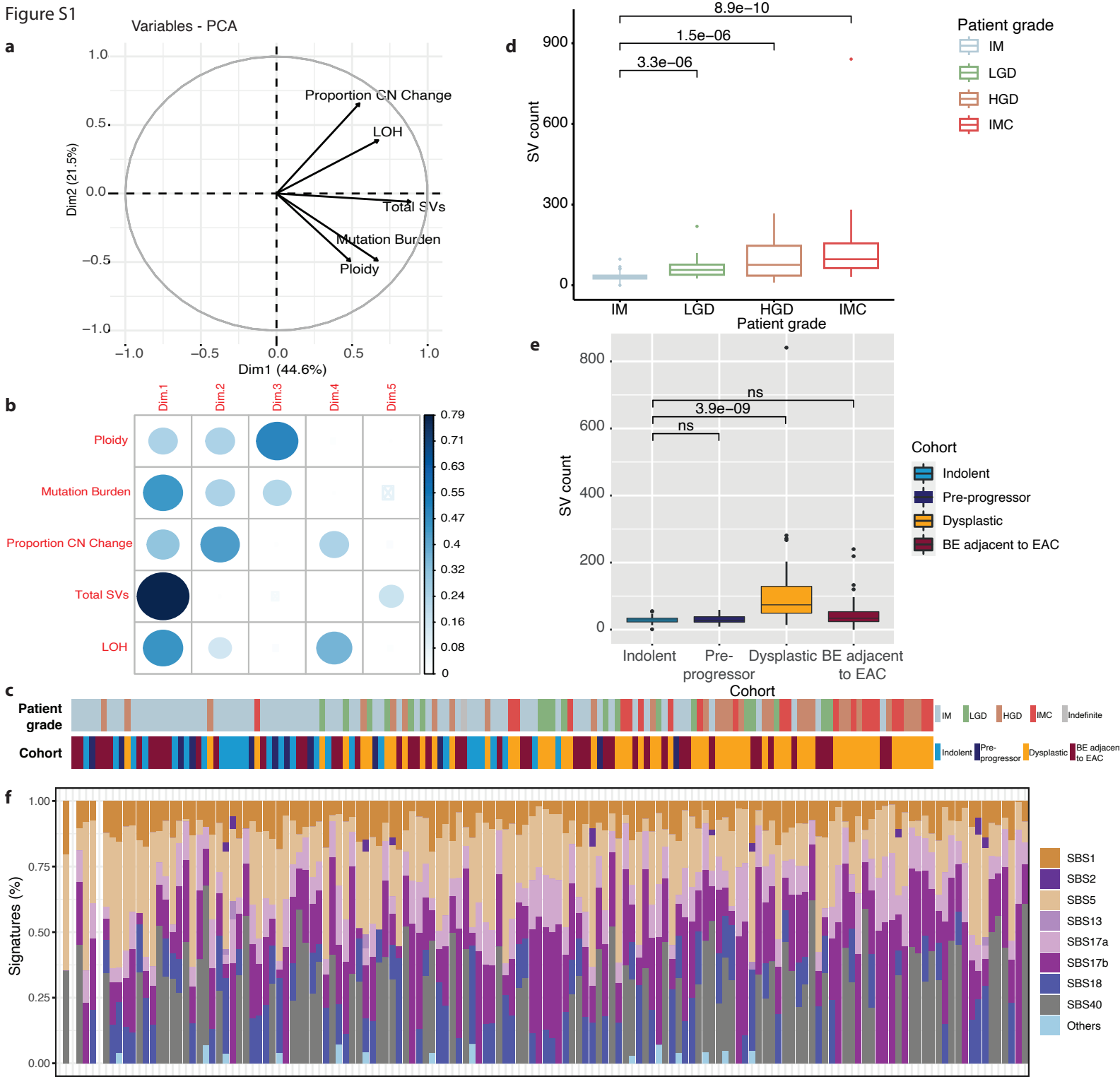

Supplement: Supplementary file 3 — Supplementary Figure 1 [file 41467_2022_28237_MOESM3_ESM.pdf]

Figure S2

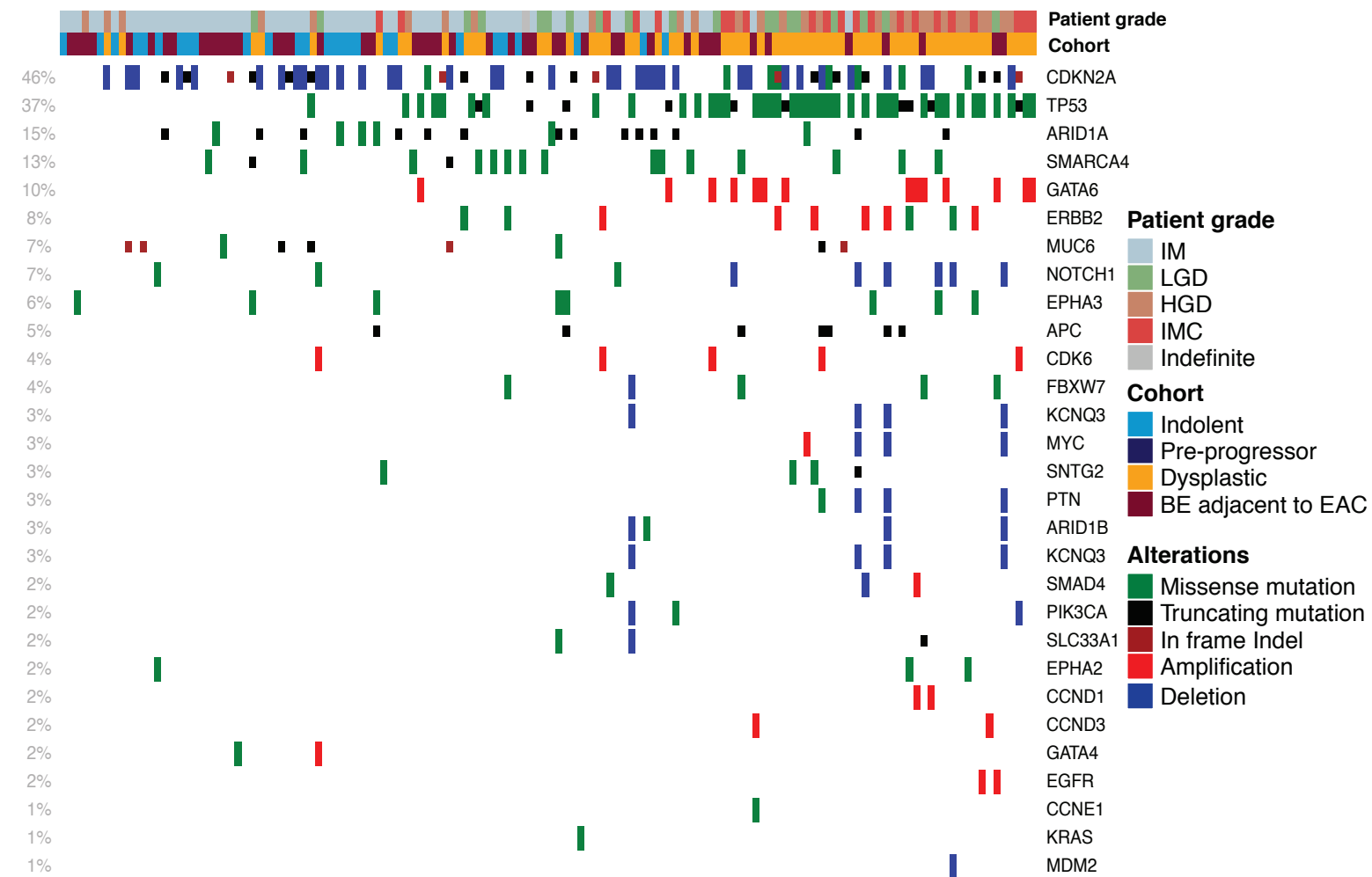

Supplement: Supplementary file 4 — Supplementary Figure 2 [file 41467_2022_28237_MOESM4_ESM.pdf]

### Figure 33

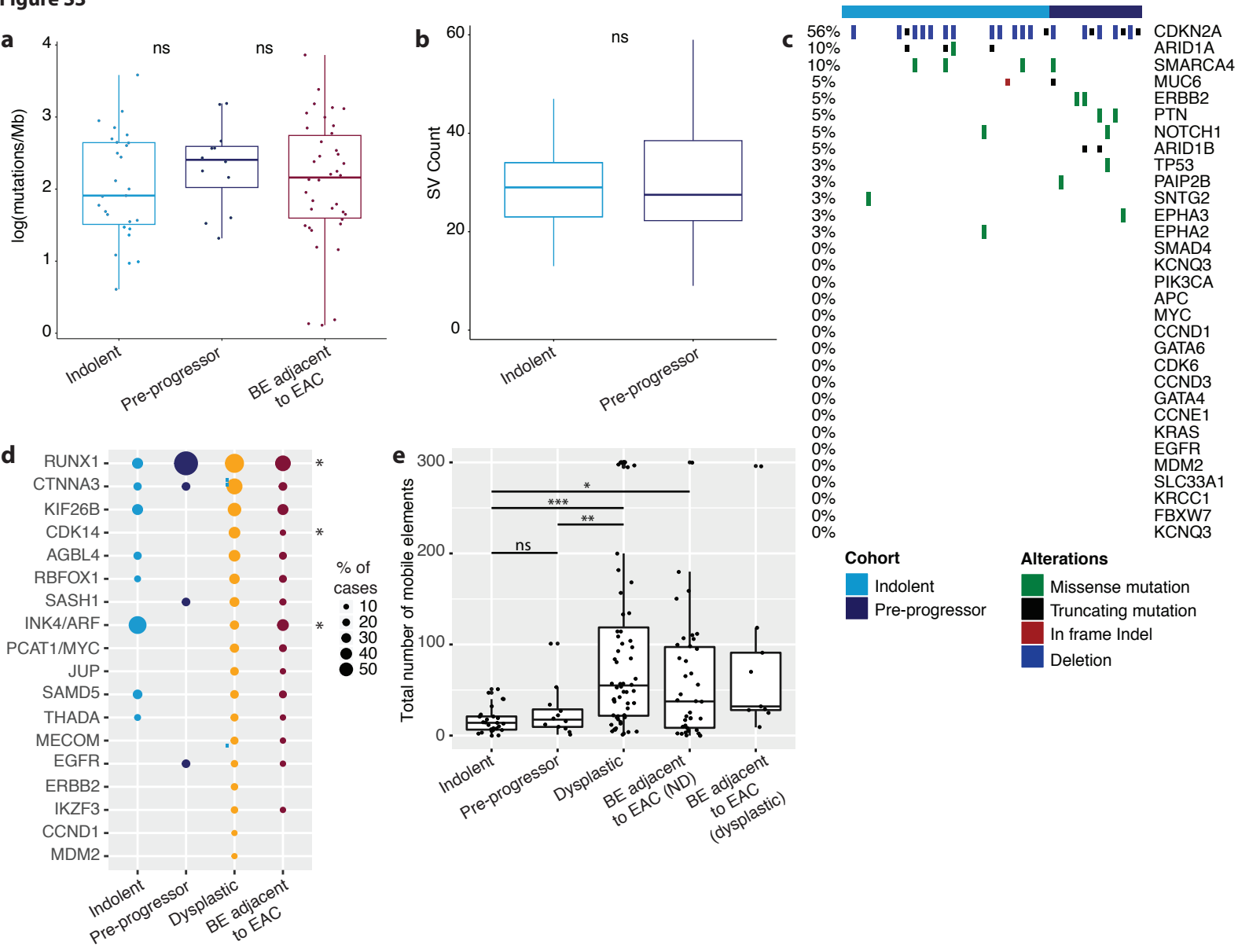

Supplement: Supplementary file 5 — Supplementary Figure 3 [file 41467_2022_28237_MOESM5_ESM.pdf]

Figure S4

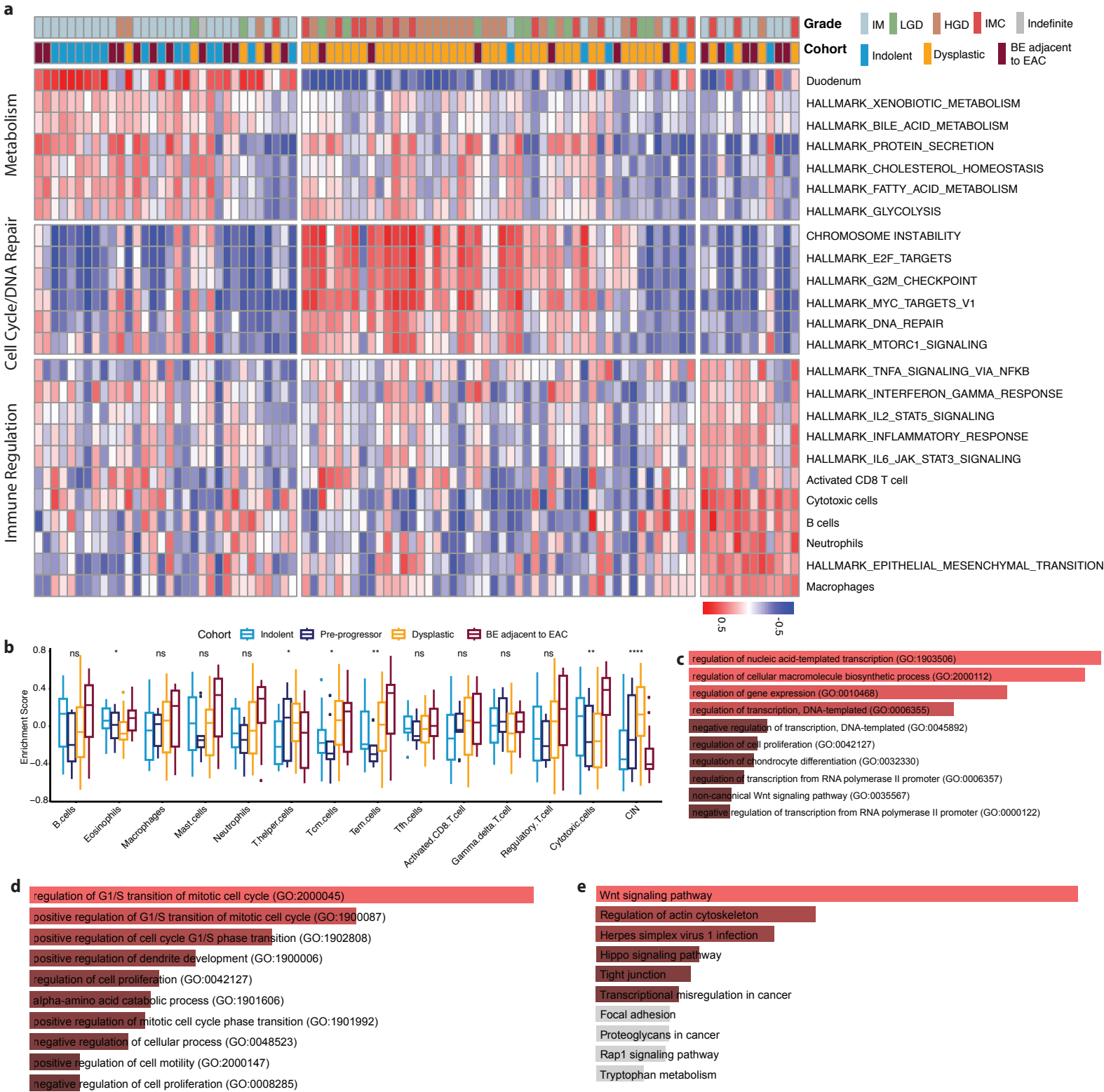

Supplement: Supplementary file 6 — Supplementary Figure 4 [file 41467_2022_28237_MOESM6_ESM.pdf]

Figure S5

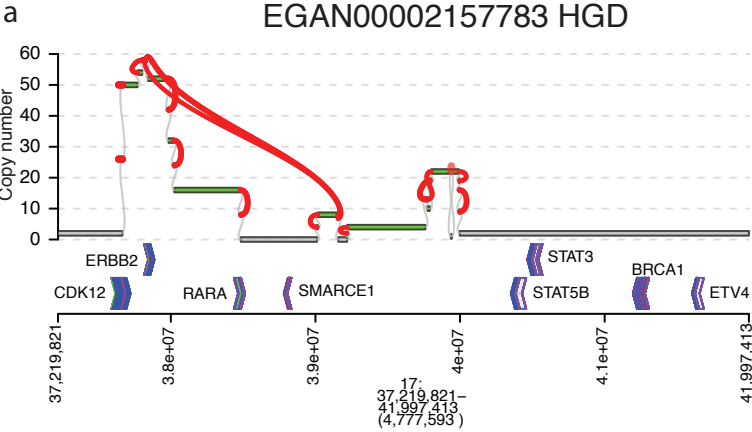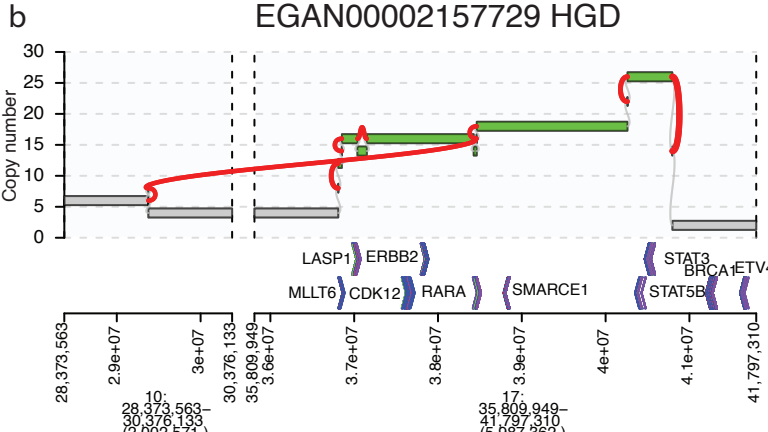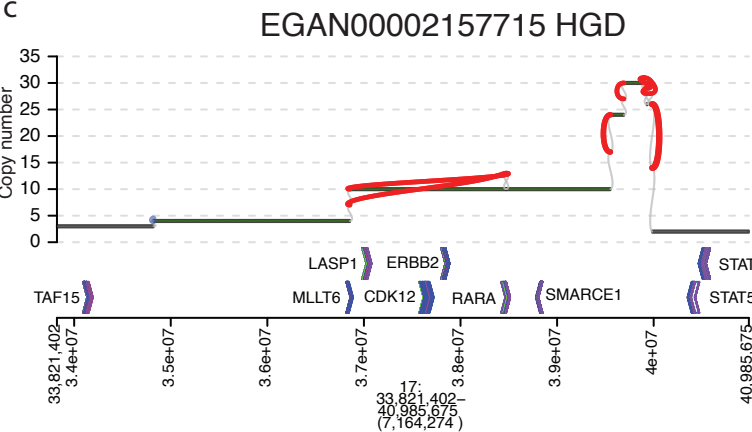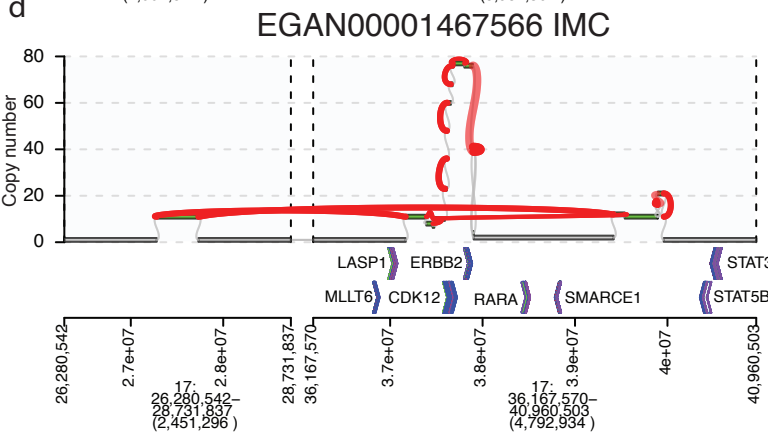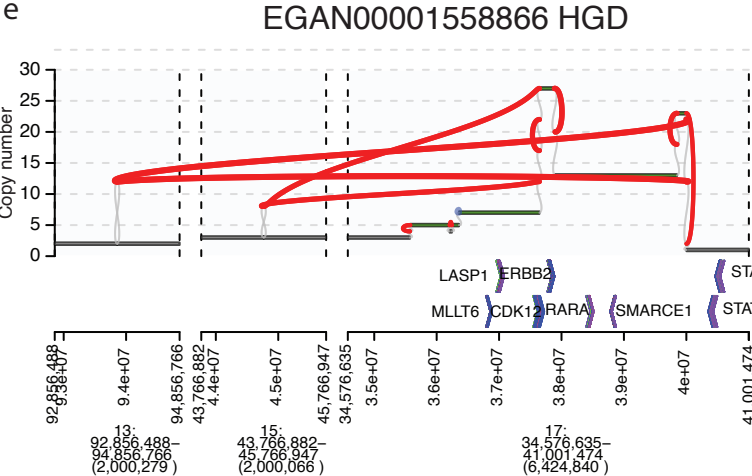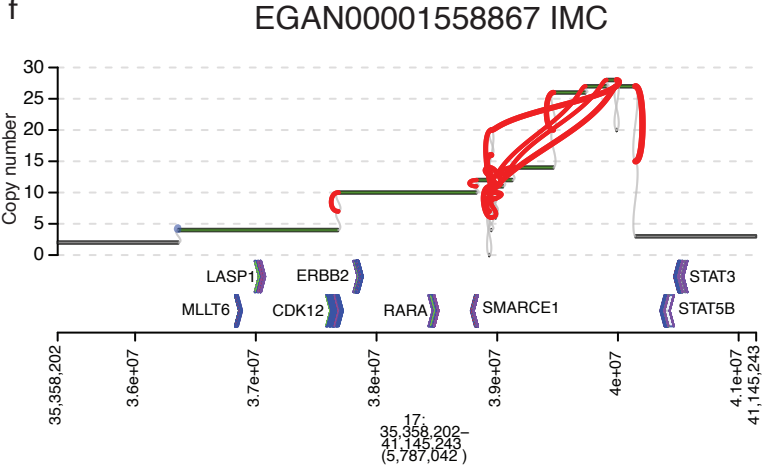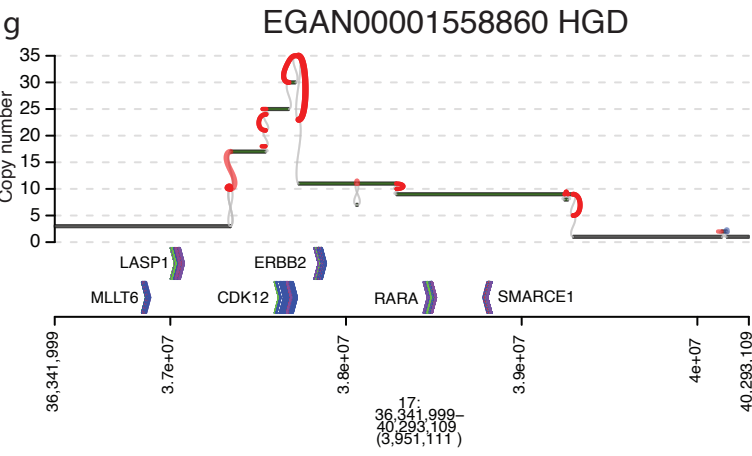

Supplement: Supplementary file 7 — Supplementary Figure 5 [file 41467_2022_28237_MOESM7_ESM.pdf]

Figure S6

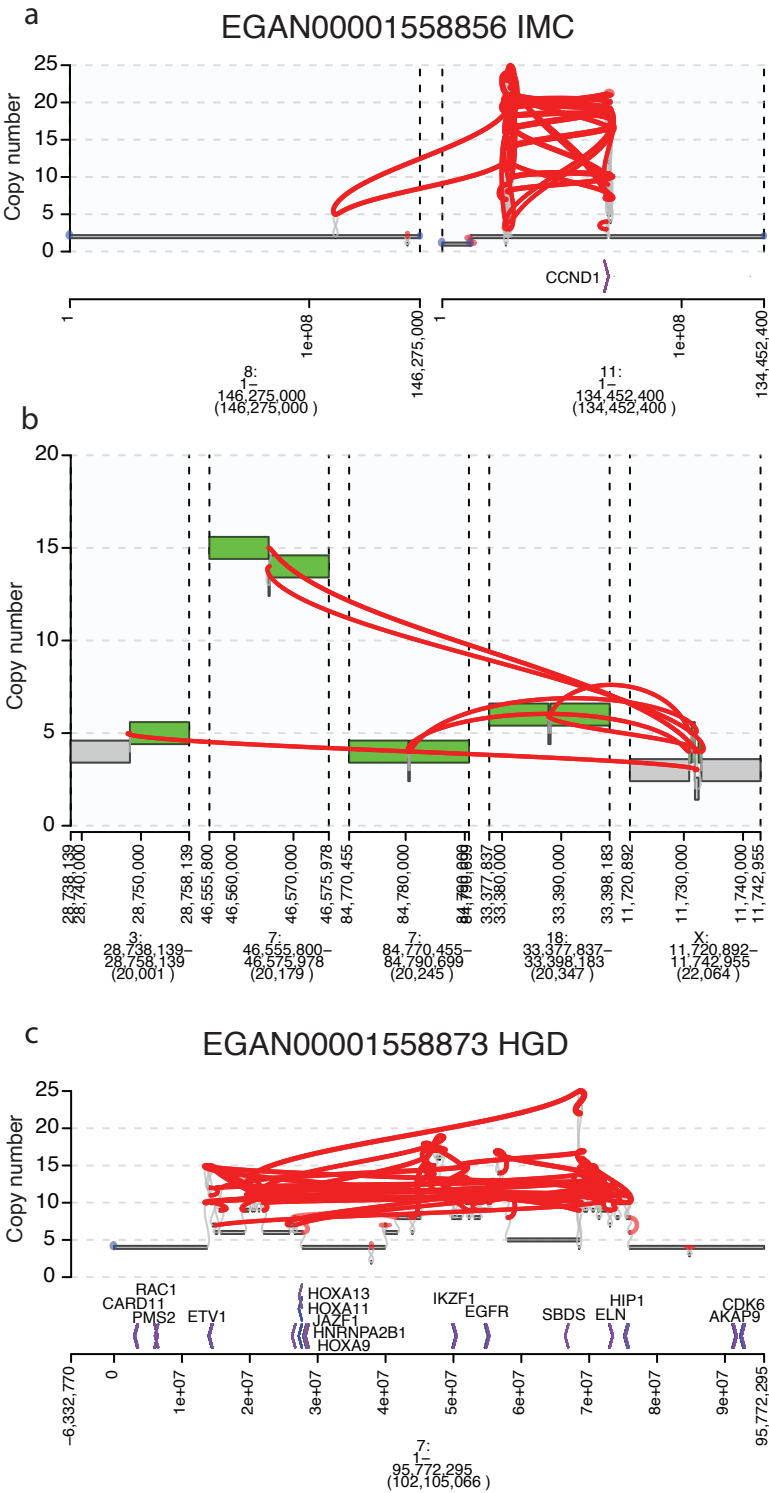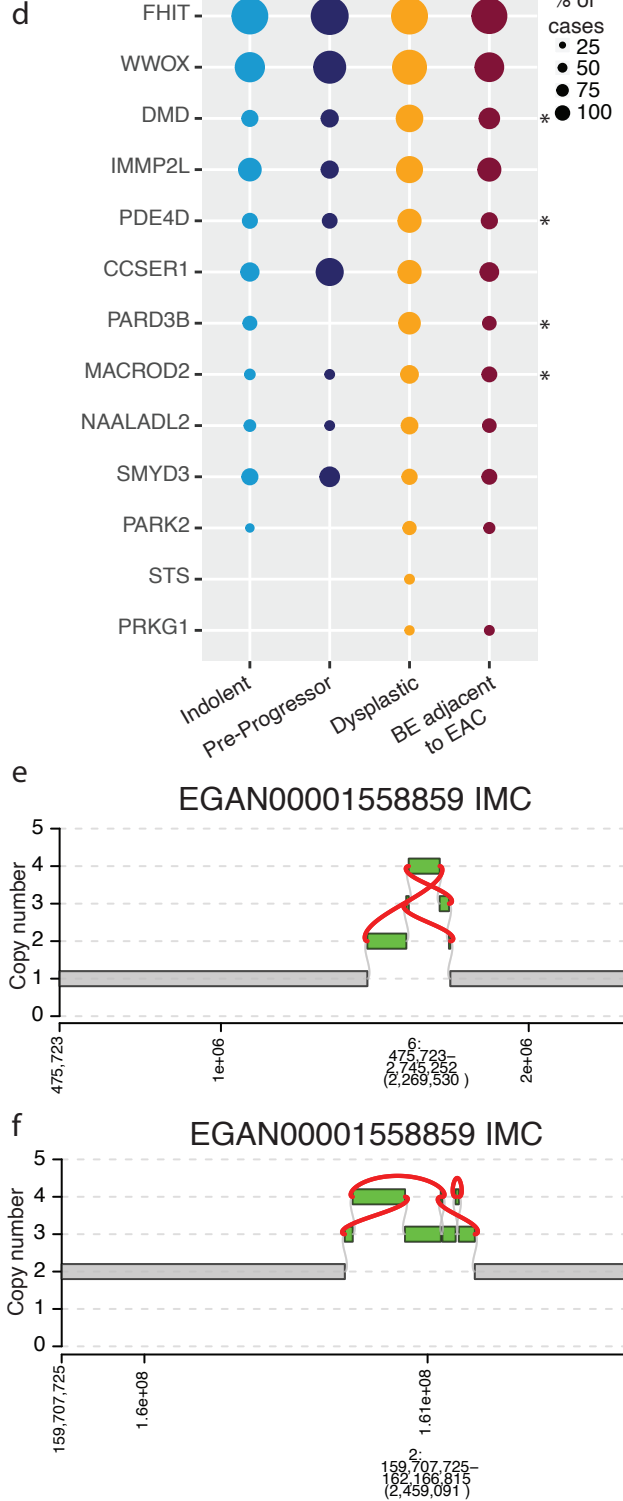

Supplement: Supplementary file 8 — Supplementary Figure 6 [file 41467_2022_28237_MOESM8_ESM.pdf]
