# Supplementary material for: Multi-omic cross-sectional cohort study of pre-malignant Barrett’s esophagus reveals early structural variation and retrotransposon activity
Source: Nat Commun. 2022 Mar 17;13:1407. doi: 10.1038/s41467-022-28237-4 (PMC8931005; doi:10.1038/s41467-022-28237-4)
Supplement: Supplementary file 11 — Reporting Summary [file 41467_2022_28237_MOESM11_ESM.pdf]

nature research

Corresponding author(s): Professor Rebecca Fitzgerald

Last updated by author(s): Mar 5, 2021

Reporting Summary

Nature Research wishes to improve the reproducibility of the work that we publish. This form provides structure for consistency and transparency in reporting. For further information on Nature Research policies, see our Editorial Policies and the Editorial Policy Checklist.

Statistics

For all statistical analyses, confirm that the following items are present in the figure legend, table legend, main text, or Methods section.

n/a	Confirmed	
☐	☒	The exact sample size (n) for each experimental group/condition, given as a discrete number and unit of measurement
☐	☒	A statement on whether measurements were taken from distinct samples or whether the same sample was measured repeatedly
☐	☒	The statistical test(s) used AND whether they are one- or two-sided<br>*Only common tests should be described solely by name; describe more complex techniques in the Methods section.*
☐	☒	A description of all covariates tested
☐	☒	A description of any assumptions or corrections, such as tests of normality and adjustment for multiple comparisons
☐	☒	A full description of the statistical parameters including central tendency (e.g. means) or other basic estimates (e.g. regression coefficient) AND variation (e.g. standard deviation) or associated estimates of uncertainty (e.g. confidence intervals)
☐	☒	For null hypothesis testing, the test statistic (e.g. F, t, r) with confidence intervals, effect sizes, degrees of freedom and P value noted<br>*Give P values as exact values whenever suitable.*
☒	☐	For Bayesian analysis, information on the choice of priors and Markov chain Monte Carlo settings
☐	☒	For hierarchical and complex designs, identification of the appropriate level for tests and full reporting of outcomes
☒	☐	Estimates of effect sizes (e.g. Cohen's d, Pearson's r), indicating how they were calculated

Our web collection on statistics for biologists contains articles on many of the points above.

Software and code

Policy information about availability of computer code

Data collection

No software used in data collection

Data analysis

No custom algorithms or software that have not already been described in the literature were used.
Software used in analysis:
R 3.4.3
FastQC 0.11.7
Burrows–Wheeler alignment (BWA-mem) 0.7.17
Picard 2.9.5
Strelka 2.0.15
Manta 0.27.2
GATK HaplotypeCaller 3.2.2
GATK ReadCountWalker 0.2.2
Battenberg 2.3.2
DPClust 2.2.5
SigProfiler 2.5.1.8
Palimpsest 1.0
ShatterSheek 0.4
NMF 0.21.0
JabBA 0.0.0.9
Impute 1.52.0
minfi 1.24.0
ChAMP 2.9.10
edgeR 3.20.9

sigproextractor 1.0.6
scipy 1.4.1
GSVA 1.26.0
Trim Galore
STAR 2.5.3a
sva package 3.20.0
GISTIC 2.0
CIRCOS
PLMIX 2.1
TraFiC 1.0
limma 3.34.9
GenomicRanges 1.30.3
MSIsensor 0.5

For manuscripts utilizing custom algorithms or software that are central to the research but not yet described in published literature, software must be made available to editors and reviewers. We strongly encourage code deposition in a community repository (e.g. GitHub). See the Nature Research guidelines for submitting code & software for further information.

Data

Policy information about availability of data

All manuscripts must include a data availability statement. This statement should provide the following information, where applicable:

- Accession codes, unique identifiers, or web links for publicly available datasets
- A list of figures that have associated raw data
- A description of any restrictions on data availability

The sequencing reads for this study have been deposited in BAM format at the European Genome-phenome Archive (EGA) under the following datasets: https://ega-archive.org/datasets/EGAD00001006349 (WGS) and https://ega-archive.org/datasets/EGAD00001006353 (RNAseq). Reads that are not used in the alignment are included to enable any reprocessing. The methylation array data has been deposited in IDAT format: https://ega-archive.org/datasets/EGAD00010001972. These are controlled access data; details on how to apply for access are available on the linked pages.

Field-specific reporting

Please select the one below that is the best fit for your research. If you are not sure, read the appropriate sections before making your selection.

☒ Life sciences ☐ Behavioural & social sciences ☐ Ecological, evolutionary & environmental sciences

For a reference copy of the document with all sections, see nature.com/documents/nr-reporting-summary-flat.pdf

Life sciences study design

All studies must disclose on these points even when the disclosure is negative.

Sample size	No sample size calculation was performed. The sample size was made as large as it could be based on the availability of suitable material for sequencing.
Data exclusions	Exclusion criteria for samples being taken forward to sequencing were pre-defined. Patients were excluded if they had progressed past the grade of interest, either before or at a later date or if they had received previous ablative treatment of their BE. Biopsies representing the independent grades could not be adjacent to cancer. Dysplastic samples for sequencing had to have a pathological cellularity of dysplasia of >30% and were included even if the dysplasia was not all the highest grade the patient was known to have. Non-dysplastic BE biopsies had to contain intestinal metaplasia. Samples with only gastric metaplasia were excluded, as were biopsies with any tumor contamination. After sequencing, data was only excluded if it failed to pass the quality control measures imposed.
Replication	No replication was performed as this was a genomic project, but all sequencing methods have previously been published.
Randomization	This was not applicable to the study. Samples were grouped based on the grade of pathology of the patient.
Blinding	All pathologists were blinded to the grade of the patient when assessing the dysplasia status and cellularity of each biopsy. No other blinding occurred because knowing the grade of the patient was integral to the analysis.

Reporting for specific materials, systems and methods

We require information from authors about some types of materials, experimental systems and methods used in many studies. Here, indicate whether each material, system or method listed is relevant to your study. If you are not sure if a list item applies to your research, read the appropriate section before selecting a response.

Materials & experimental systems

n/a	Involved in the study
☒	☐ Antibodies
☒	☐ Eukaryotic cell lines
☒	☐ Palaeontology and archaeology
☒	☐ Animals and other organisms
☐	☒ Human research participants
☒	☐ Clinical data
☒	☐ Dual use research of concern

Methods

n/a	Involved in the study
☒	☐ ChIP-seq
☒	☐ Flow cytometry
☒	☐ MRI-based neuroimaging

Human research participants

Policy information about studies involving human research participants

Population characteristics

The only significant differences between the groups were the smoking status between indolent and progressed groups and the pre-progressors were significantly younger than the indolent group. There were no significant differences between age or sex between the groups. Information about the human research participants can be found in Table 1 of the manuscript

Recruitment

Samples used for this study came from a biobank of sample from participants who were recruited via 3 trials. Patients were invited to take part if they were known to have Barrett's esophagus and had been referred for endoscopy in the region. The East of England has a predominantly Caucasian population and this is reflected in the patient demographics of the samples. The self-selection bias is reduced in these studies as these patients were already undergoing endoscopy/surgery and the trials did not require additional endoscopies. Samples were collected during standard visits.
All Barrett's esophagus (BE) patients were selected from our Cell Determinants Biomarker (REC no. 01/149), BEST2 (REC no. 10/H0308/71) and OCCAMS (Rec. no. 10-H0305-1) trials. Cell Determinants Biomarker is an observational trial which focuses on determining biomarkers to identify patients with BE who have a higher risk of progression to esophageal cancer. BEST2 is a case:control observational study using the CytospongeTM to test for diagnosing BE. OCCAMS is an observational study to determine the molecular drivers of EAC

Ethics oversight

Ethical approval was from the East of England-Cambridge Central Research Ethics Committee. Tissue was obtained with written, informed patient consent. All relevant ethical regulations were correctly followed and samples were fully anonymized.

Note that full information on the approval of the study protocol must also be provided in the manuscript.
